# Blocking variant surface glycoprotein synthesis alters endoplasmic reticulum exit sites/Golgi homeostasis in *Trypanosoma brucei*

**DOI:** 10.1111/tra.12561

**Published:** 2018-04-06

**Authors:** Cher‐Pheng Ooi, Terry K. Smith, Eva Gluenz, Nadina Vasileva Wand, Sue Vaughan, Gloria Rudenko

**Affiliations:** ^1^ Department of Life Sciences Imperial College London London UK; ^2^ BSRC, School of Biology University of St. Andrews St. Andrews UK; ^3^ Sir William Dunn School of Pathology University of Oxford Oxford UK; ^4^ Department of Biological and Medical Sciences Oxford Brookes University Oxford UK

**Keywords:** ER exit site, Golgi biogenesis, membrane metabolism, secretory pathway, *Trypanosoma brucei*, variant surface glycoprotein

## Abstract

The predominant secretory cargo of bloodstream form *Trypanosoma brucei* is variant surface glycoprotein (VSG), comprising ~10% total protein and forming a dense protective layer. Blocking VSG translation using Morpholino oligonucleotides triggered a precise pre‐cytokinesis arrest. We investigated the effect of blocking VSG synthesis on the secretory pathway. The number of Golgi decreased, particularly in post‐mitotic cells, from 3.5 ± 0.6 to 2.0 ± 0.04 per cell. Similarly, the number of endoplasmic reticulum exit sites (ERES) in post‐mitotic cells dropped from 3.9 ± 0.6 to 2.7 ± 0.1 eight hours after blocking VSG synthesis. The secretory pathway was still functional in these stalled cells, as monitored using Cathepsin L. Rates of phospholipid and glycosylphosphatidylinositol‐anchor biosynthesis remained relatively unaffected, except for the level of sphingomyelin which increased. However, both endoplasmic reticulum and Golgi morphology became distorted, with the Golgi cisternae becoming significantly dilated, particularly at the trans‐face. Membrane accumulation in these structures is possibly caused by reduced budding of nascent vesicles due to the drastic reduction in the total amount of secretory cargo, that is, VSG. These data argue that the total flux of secretory cargo impacts upon the biogenesis and maintenance of secretory structures and organelles in T. brucei, including the ERES and Golgi.

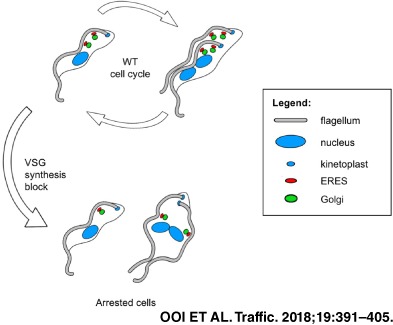

## 1 INTRODUCTION

The bloodstream form (BF) of *Trypanosoma brucei* is an unusual unicellular eukaryote, in that the bulk of its secretory cargo is comprised of a single protein: variant surface glycoprotein (VSG).^1^ *T. brucei* is the causative agent of human African trypanosomiasis and related diseases in livestock.^2^ While multiplying within the bloodstream of the host, each trypanosome is coated with an extremely dense layer of VSG protecting the cell from lysis by the alternative pathway of the complement system. As the predominant component of the trypanosome surface, VSG is by far the most abundant protein in the cell (~10% total). As VSG is extracellular, it progresses through the trypanosome secretory pathway, and therefore, makes up the vast majority of the secretory cargo of BF *T. brucei.* 3 The approximately 10^7^ VSG molecules per cell are extended homodimers, which are attached to the cell surface via glycosylphosphatidylinositol (GPI)‐anchors.^4^ This allows the VSG molecules to have an extremely high density of cell‐surface packing, yet diffuse freely over the plasma membrane.^5^, ^6^

The secretory pathway of *T. brucei* is unusual, in that it has reduced complexity compared with other eukaryotes.^3^, ^7^, ^8^ In mammalian cells there are dozens of Golgi bodies per cell,^9^ while in plant cells there are between 25 and 600 Golgi stacks per cell depending upon plant cell type.^10^ In the baker's yeast *Saccharomyces cerevisiae* the “classic” Golgi stack structure is absent, and there are about 20 dispersed cisternae per cell.^11^ In contrast, in procyclic *T. brucei* present in the insect vector, there is normally only one Golgi stack per cell.^12^ These cells are coated in procyclin protein, which is less abundant than VSG, comprising about 1% total protein.^13^ In BF *T. brucei* there are an increased number of Golgi bodies, with typically about 60% to 70% of cells in G1 with 2 Golgi bodies, rising to at least 4 Golgi in more than 60% of cells that have completed mitosis, but not yet initiated a cleavage furrow.^8^, ^14^ The increased number of Golgi bodies in bloodstream compared with procyclic form *T. brucei* could possibly be an adaptation allowing the cell to accommodate the vast amounts of VSG (10‐fold higher than procyclin) that need to be processed through the secretory pathway to the cell surface in this life cycle stage. Hence, this small number of very active Golgi bodies per cell makes *T. brucei* an amenable system to investigate Golgi biogenesis.

Different models have been proposed for Golgi biogenesis, either relying upon *de novo* synthesis, or duplication of a pre‐existing Golgi template.^15^ In *T. brucei* a hybrid of the two models appears to exist, whereby the new Golgi is formed *de novo* at a fixed distance from the old Golgi,^12^ but requiring components from the old Golgi.^16^ However, in addition to these two models, studies in yeast have postulated that continuing vesicular traffic is essential for maintaining correct Golgi structure.^15^, ^17^ How the amount of secretory traffic affects Golgi maintenance and structure in *T. brucei* is unclear. We, therefore, attempted to investigate this by blocking synthesis of the major secretory cargo of the BF trypanosome, VSG.

VSG is central to *T. brucei* pathogenicity.^18^, ^19^ Surprisingly, VSG is essential even in vitro in BF *T. brucei*, and blocking its synthesis with RNAi results in cells arresting at a very precise cell‐cycle stage, immediately before cell division.^20^ As there is no re‐initiation of S‐phase in these stalled cells, this is compatible with VSG synthesis being monitored during the cell cycle, and a precise cell cycle checkpoint triggered in the absence of its synthesis.^21^ However it is unclear what aspect of VSG (ie, RNA or protein) is being “sensed” to trigger this cell cycle checkpoint.

In order to obtain insight into this “sensing” mechanism, we attempted to block VSG synthesis at the level of translation, while leaving *VSG* mRNA intact. In order to investigate the connection between amount of secretory cargo and homeostasis of secretory organelles in BF *T. brucei*, we emptied the secretory pathway of its predominant cargo (VSG) in an inducible fashion. We characterised the secretory structures and organelles in cells where VSG synthesis had been inducibly blocked. We show that blocking VSG synthesis at the level of translation produces an equivalent arrest to that derived after ablating *VSG* mRNA. This argues that VSG synthesis or transport is being “sensed” rather than VSG transcript. In addition, stopping VSG synthesis leads to a reduction in the number of endoplasmic reticulum exit sites (ERES) and Golgi. Both the endoplasmic reticulum (ER) and Golgi showed distortions in their morphology, with distended cisternae particularly at the trans‐face of the Golgi. This specific membrane accumulation argues that post‐Golgi secretory vesicles only bud off from these structures if there is adequate cargo to fill them. These results support a model that there is a direct connection between amount of secretory cargo and secretory organelle homeostasis in *T. brucei.*

## 2 RESULTS

### 2.1 Morpholino mediated block of VSG mRNA translation triggers a pre‐cytokinesis arrest

**Figure 1:**
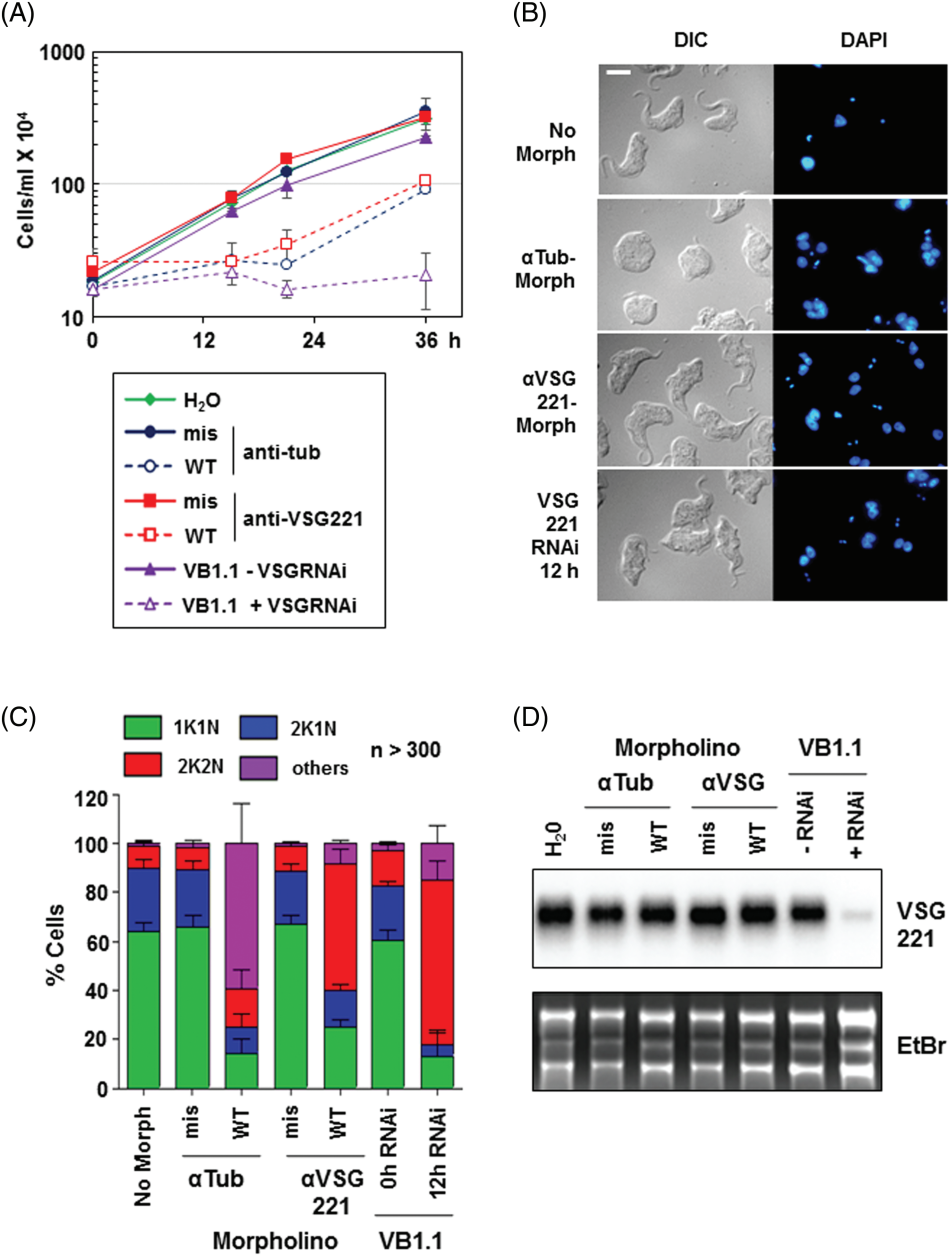
Blocking translation of VSG mRNA using antisense Morpholino oligonucleotides triggers a specific pre‐cytokinesis cell cycle arrest. A, Perturbation of VSG synthesis with Morpholinos results in a growth arrest. Growth curves of Trypanosoma brucei after electroporation with anti‐tubulin (tub) or anti‐*VSG221* Morpholinos with water (H_2_O) shown as a negative control. Morpholinos were designed against either the WT sequences or had mismatches (mis) introduced to disrupt base‐pairing with mRNA. In comparison, T. brucei VB1.1 in the presence (+) or absence (−) of tetracycline to induce *VSG221* RNAi for the time indicated in hours (h) is shown. Error bars indicate the SD for biological replicates (*n* = 4 for anti‐tubulin Morpholinos and H_2_O, n = 3 for anti‐VSG Morpholinos and n = 2 for *VSG221* RNAi). The scale bar indicates 5 μM. B, Microscopy analysis of T. brucei 12 hours after electroporation of anti‐tubulin Morpholinos (αTub‐Morph) or anti‐*VSG221* Morpholinos (αVSG221‐Morph) with no Morpholinos (water) shown as a control. Trypanosoma brucei VB1.1 cells with *VSG221* RNAi induced for 12 hours (h) are included for comparison. Images of cells visualised with differential interference contrast (DIC) or with DNA stained with DAPI (blue) are shown. C, Cell cycle analysis shows that blocking VSG synthesis with Morpholinos is comparable to induction of *VSG* RNAi. Cells were karyotyped 12 hours after electroporation of anti‐tubulin (αTub) or anti *VSG221* (αVSG221) with water (No Morph) serving as a negative control. Anti‐sense Morpholinos were either against the WT sequence or mis‐matched (mis). The percentage (%) of cells at the 1K1N, 2K1N or 2K2N stage of the cell cycle is shown, with “others,” including multi‐nucleated cells which had reinitiated S‐phase. In comparison, T. brucei VB1.1 before or after the induction of *VSG221* RNAi for 12 hours (h) is shown. Error bars indicate the SD from multiple biological replicates (*n* = 4 for anti‐tubulin Morpholinos and the no Morpholino control, *n* = 3 for anti‐*VSG221* Morpholinos and *n* = 2 for T. brucei VB1.1). (*n* > 300 cells were counted for each treatment in each biological replicate). D, Electroporation of anti‐*VSG221* Morpholino oligonucleotides does not lead to reduction in *VSG221* mRNA. Northern blot analysis shows *VSG221* transcript 12 hours after cells were transfected with either anti‐tubulin (αTub) or anti‐VSG221 (αVSG) antisense Morpholinos. In parallel, water (H_2_O) was electroporated as a negative control. RNA from T. brucei VB1.1 after the induction of *VSG221* RNAi for 12 hours is shown in comparison. An image of the gel stained with ethidium bromide (EtBr) is shown to indicate loading

We have previously shown that the RNAi mediated ablation of *VSG* mRNA in BF *T. brucei* triggers a specific pre‐cytokinesis cell cycle arrest, which is characterised by the absence of re‐initiation of S phase in the stalled cells.^20^ In order to determine if this unique cell cycle checkpoint was sensed at the protein or the RNA level, we attempted to block VSG synthesis by preventing its translation without affecting *VSG* mRNA levels. We investigated this using Morpholino antisense oligonucleotides (Morpholinos) designed to bind the region downstream of and including the start codon of *VSG221* mRNA, with Morpholinos targeting α‐tubulin mRNA serving as a positive control (Figure S1A, Supporting Information). As a negative control, anti‐VSG and anti‐tubulin Morpholinos were designed, each including 5 mismatched nucleotides which would disrupt Morpholino binding to the target mRNA (Figure S1A). BF *T. brucei* HNI (221+) cells expressing VSG221 were transfected with both anti‐VSG and anti‐tubulin Morpholinos.^22^ As expected for a perturbation disrupting VSG synthesis, transfection of anti‐*VSG221* Morpholinos resulted in *T. brucei* arresting in cell growth immediately after the transfection, comparable to as observed after the induction of *VSG221* RNAi in *T. brucei* VB1.1 (Figure 1A).^20^ Transfection of cells with mismatched anti‐*VSG221* Morpholinos did not trigger this growth arrest. As a control, disruption of tubulin translation also resulted in the expected perturbation of cell growth. The cell cycle arrest phenotype triggered by transfection with Morpholinos was transient, with cells recovering 21 hours after electroporation with anti‐*VSG221* Morpholinos and after 36 hours for cells transfected with anti‐tubulin Morpholinos (Figure 1A). In contrast, the cell cycle arrest induced by *VSG221* RNAi was relatively more stable.

*Trypanosoma brucei* transfected with the anti‐tubulin Morpholinos become spherical and were multi‐nucleated after 12 hours, due to re‐initiation of S‐phase in the stalled cells (Figure 1B). This is consistent with the FAT phenotype generated after blocking tubulin synthesis using anti‐tubulin RNAi.^23^ In contrast, there was no change in cellular morphology in cells where either no Morpholinos were transfected or mismatched anti‐tubulin Morpholinos were used (Figure 1A; Figure S1B). As expected, blocking VSG221 synthesis through the induction of *VSG* RNAi resulted in cells which had stalled at a precise post‐mitotic stage characterised by 2 nuclei (N) and 2 kinetoplasts (K), but before initiation of a cleavage furrow (Figure 1B,C).^20^ Cells observed 12 hours after transfection of anti‐*VSG221* Morpholinos arrested in a similar fashion to those where *VSG* RNAi had been induced (Figure 1B,C).

Transfection of *T. brucei* with anti‐tubulin Morpholinos resulted in the majority of the cells (55%) stalling before cell division as multi‐nucleated cells that had undergone re‐initiation of S‐phase (“others” in Figure 1C). In contrast, although the induction of *VSG* RNAi also prevented cell division, there was no re‐initiation of S phase, resulting in an accumulation of 2K2N cells to 67% of the population (Figure 1C).^20^ Essentially equivalent results (52% 2K2N) were obtained after transfection with anti‐*VSG221* Morpholinos. Northern blot analysis showed that although induction of *VSG221* RNAi in *T. brucei* VG1.1 resulted in the ablation of *VSG221* mRNA to nearly undetectable levels after 12 hours, transfection of the various Morpholinos, as expected did not affect the level of *VSG221* transcript (Figure 1D). It therefore appears that with regards to this checkpoint response, it is levels of VSG protein, VSG protein synthesis or VSG transport that are “sensed” during trypanosome progression through the cell cycle, rather than the *VSG* mRNA itself.

### 2.2 Blocking synthesis of VSG results in a reduced number of ER exit sites and Golgi

As the most abundant protein in BF *T. brucei*, VSG makes up the vast majority of the total secretory cargo. Along with other secretory cargo, including various surface receptors, VSG is recruited to COPII vesicles at the ERES characterised by TbSec23/ TbSec24 heterodimers,^24^ and is subsequently transported through the Golgi cisternae to eventually be transported to the cell surface by secretory vesicles.^25^, ^26^ Each BF trypanosome typically has two ERES:Golgi junctions, which are replicated as the cell progresses through the cell cycle.^14^ This culminates in cells with normally about four ERES and Golgi immediately after mitosis.^8^, ^14^ We, therefore, investigated if blocking VSG synthesis, and therefore, emptying the secretory pathway of a significant amount of its cargo, affected the total number of ER exit sites and Golgi.

**Figure 2:**
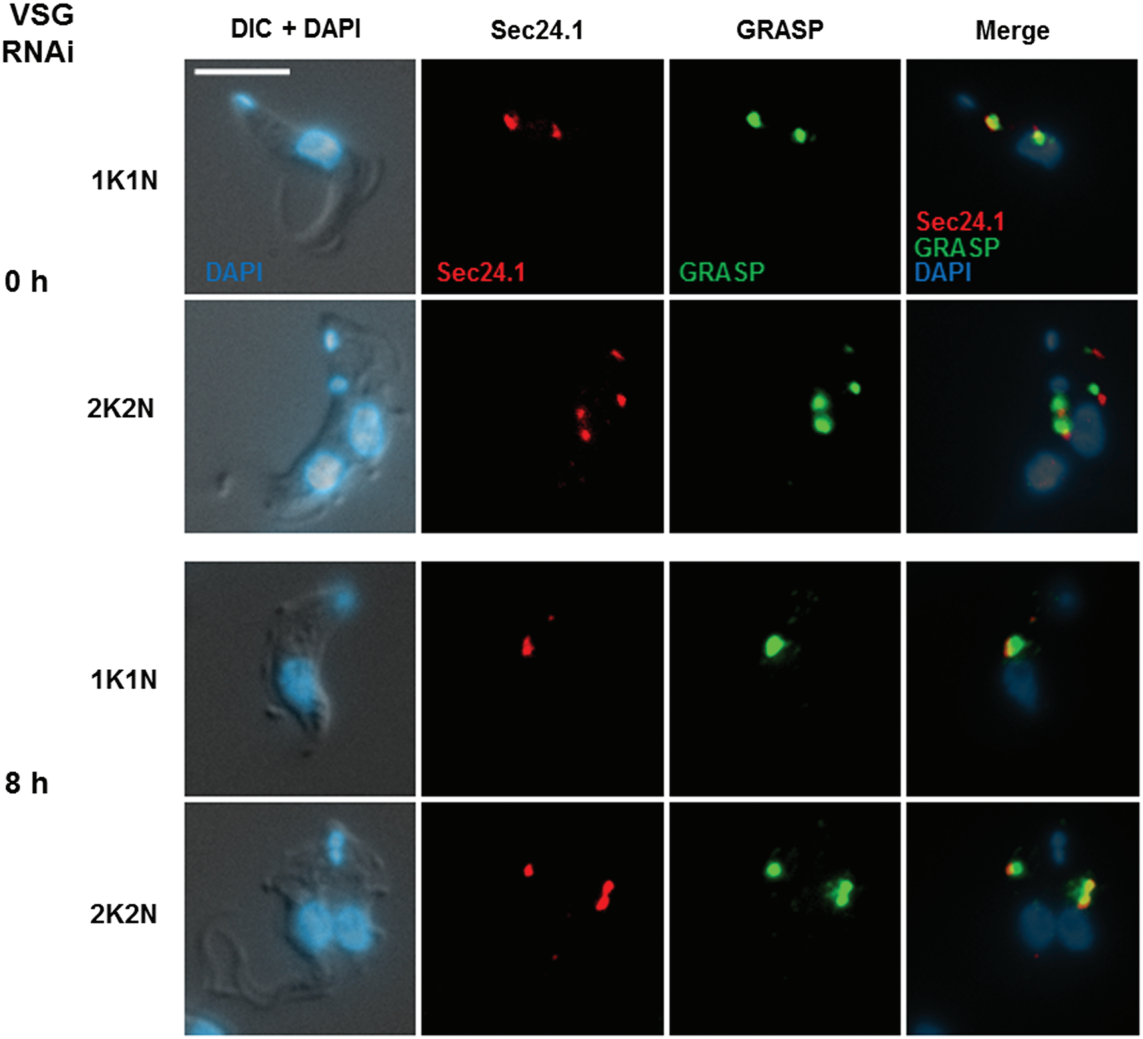
Microscopy analysis of ERES and Golgi in T. brucei stalled after the induction of a VSG synthesis block. Trypanosoma brucei cells were visualised after the induction of *VSG221* RNAi for 0 or 8 hours (h), where cells in either the 1K1N or 2K2N stage of the cell cycle are compared. The ERES was visualised using Ty1 epitope tagged TbSec24.1 and the Golgi were visualised using an antibody against the TbGRASP Golgi marker. Representative images of T. brucei SL221TbSec24.1::Ty cells probed with antibodies for Ty1 (red) or GRASP (green) are shown. Images are a merge of these 2 signals in addition to the DNA stain DAPI (blue) and the outline of the cells as visualised using differential interference contrast (DIC). Scale bar is 5 μM

We investigated this in BF *T. brucei* SL221 TbSec24.1::Ty1 where the ER exit sites were visualised using Ty1 epitope tagged TbSec24.1,^24^ while the Golgi were visualised with an antibody against the GRASP marker for the mid‐Golgi.^12^ The localisation of TbSec23.2 to the ERES has previously been demonstrated to a high degree of resolution using immunogold electron microscopy.^24^ In order to confirm that TbSec24.1 indeed colocalised with TbSec23.2, we epitope tagged TbSec23.2 with HA in the BF *T. brucei* SL221TbSec24.1::Ty1 cell line. Simultaneous detection of epitope tagged TbSec24.1 and TbSec23.2 showed good overlap of both proteins when probing for both the Ty1 and HA epitopes simultaneously using immunofluorescence microscopy (Figure S2A). Quantitation of the overlap (Figure 2B) showed that TbSec23.2 had a mean displacement from TbSec24.1 of 0.03 ± 0.3 μM (*n* = 25 1K1N cells measured with error in SD), confirming that TbSec24.1 colocalises with TbSec23.2 in the ERES as previously reported.^24^

As expected, immunofluorescence microscopy analysis showed that the ERES and Golgi were located immediately adjacent to each other as non‐overlapping spots within the cell,^12^, ^14^, ^24^ and this subcellular location was not disrupted after blocking VSG synthesis for 8 hours (Figure 2). This allowed quantitation of the number of ERES and Golgi after the induction of a VSG synthesis block.

**Figure 3:**
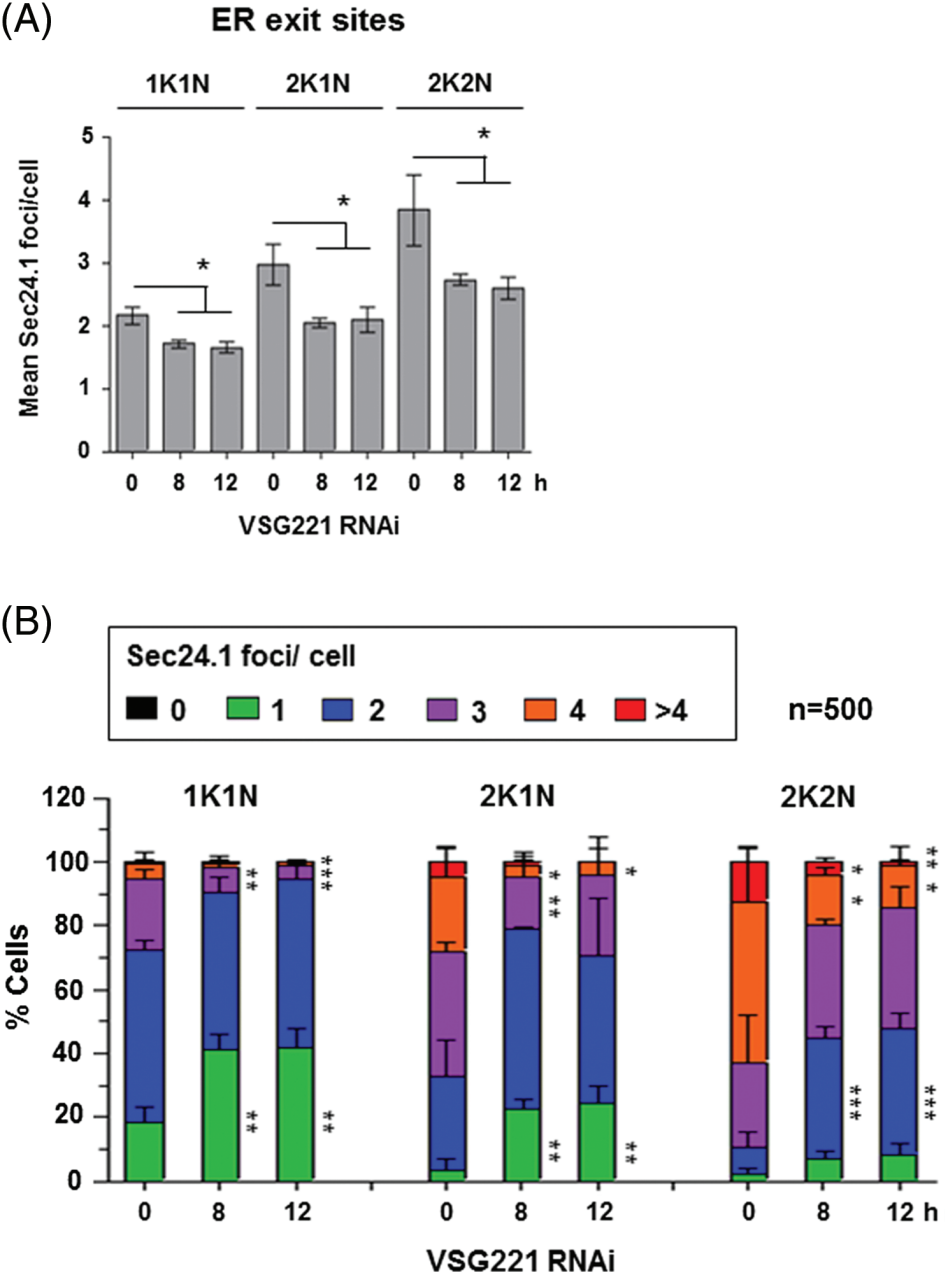
The number of ER exit sites is reduced after induction of a VSG synthesis block. A, ER exit sites were quantitated by monitoring Ty1 epitope tagged Sec24.1 in T. brucei SL221TbSec24.1::Ty1 cells using an anti‐Ty1 antibody. Mean TbSec24.1 foci per cell were counted in cells where *VSG221* RNAi had been induced for the time indicated in hours (h). Data are grouped according to cell cycle stage analysed (1K1N, 2K1N or 2K2N). Error bars show the SD from 3 biological replicates (*n* ~500 cells per time point for each replicate). Significance of the difference (*P < .05) was determined from paired t‐tests compared with uninduced cells. B, Data in panel (A) presented as the percentage (%) of cells grouped according to the number of TbSec24.1 foci per cell after induction of *VSG221* RNAi for the time indicated in hours (h). Error bars show the SD from 3 biological replicates (*n* ~500 cells per time point for each replicate). The significance of the difference (*P < .05, **P < .01, ***P < .001) for changes to the percentage of cells with a given number of foci was calculated using one‐way anova followed by Tukey post‐hoc comparing the 0, 8 and 12 h time points within each cell cycle stage (1K1N, 2K1N, 2K2N)

In cells where VSG synthesis had been blocked for 8 to 12 hours, there was a significant reduction in the number of ERES (Figure 3). In *T. brucei,* the kinetoplast (K), that is, the mitochondrial DNA, replicates before the nuclear (N) DNA, meaning that these can be used to follow trypanosome progression through the cell cycle.^27^ The mean number of ERES foci per cell decreased in cells where VSG synthesis was blocked for 8 hours, and particularly in replicating cells (2K1N and 2K2N cells). Here, the number of ERES foci in S‐phase cells (2K1N) was significantly reduced from 3.0 ± 0.3 to 2.0 ± 0.1 per cell, and in post‐mitotic cells (2K2N) from 3.9 ± 0.6 to 2.7 ± 0.1 per cell after blocking VSG synthesis for 8 hours (*P* < .05). The mean number of ERES per cell further remained relatively unchanged after 12 hours induction of *VSG* RNAi.

There was also a significant change in the distribution of the number of ERES foci at the different stages of the *T. brucei* cell cycle. At the G1 cell cycle stage (1K1N), including both non‐dividing and very early dividing cells, the most significant change was in the reduction in cells with 3 ERES foci from 22.3% ± 3.2% to 8.0% ± 3.1% after 8 hours *VSG* RNAi (*P* < .01), with a corresponding increase in cells with one ERES focus from 18.4% ± 4.8% to 41.5% ± 6.4% (*P* < .01). At the phase (2K1N) stage, there was a significant reduction (*P* < .05) in cells with 4 ERES foci from 23.3% ± 9.2% to 3.6% ± 4.1%, and a corresponding increase in cells with only one focus from 3.6% ± 3.3% to 22.5% ± 3.0% at 8 hours (*P* < .01). At the G2/M (2K2N) stage there was a significant decrease (*P* < .05) in the percentage of cells with 4 foci (50.4% ± 17.3% to 16.0% ± 2.3% or more than 4 foci (12.4% ± 4.3% to 3.9% ± 1.4% at 8 hours), with a corresponding very significant increase (*P* < .001) in cells with 2 foci (8.4% ± 4.7% to 38.1% ± 3.3% at 8 hours). These results are all compatible with the hypothesis that the number of ERES foci within the cell is subject to requirement, and is dictated by the amount of secretory cargo.

**Figure 4:**
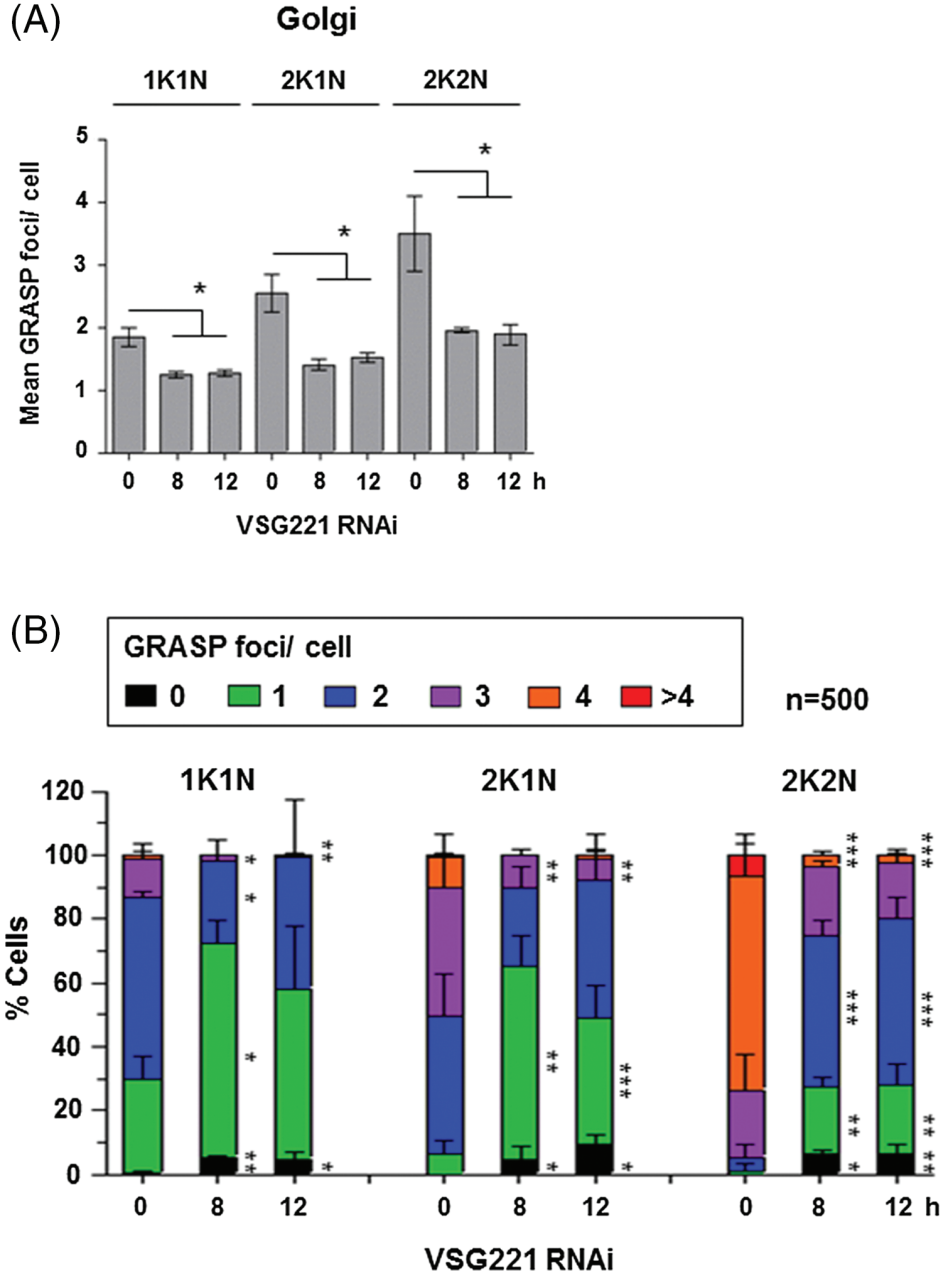
The number of Golgi is reduced in cells arrested after the induction of *VSG221* RNAi. A, The number of Golgi were quantitated using an antibody against the GRASP Golgi marker in Trypanosoma brucei SL221TbSec24.1::Ty1 cells which were also co‐stained with a Ty1 antibody to visualise the TbSec24.1 marker for the ERES. Only Golgi foci associated with TbSec24.1 were counted to avoid counting of “phantom Golgi”.^14^ *VSG221* RNAi was induced for the time indicated in hours (h). Data are grouped according to cell cycle stage (1K1N, 2K1N, 2K2N). Error bars show the SD from 3 biological replicates (*n* ~500 cells per time point per biological replicate). The significance of the difference with uninduced cells (*P < .05) was determined using paired *t* tests. B, Data in panel (A) presented as the percentage of cells in each cell cycle stage with a given number of Golgi foci. Error bars show the SD from 3 biological replicates (*n* ~500 cells per time point per replicate). Significance of the difference (*P < .05, **P < .01, ***P < .001) of the change in cells with a given number of foci was determined using one‐way anova followed by Tukey post‐hoc comparing the different points of *VSG* RNAi within each cell cycle stage

A similar analysis of the number of Golgi bodies was performed after the induction of a VSG synthesis block. The Golgi were defined as GRASP positive foci near the flagellar attachment zone, and in close proximity to the ERES as visualised with TbSec24.1.^14^ The mean number of GRASP foci per cell decreased significantly (*P* < .05) after induction of *VSG* RNAi for 8 or 12 hours (Figure 4A). After 8 hours *VSG* RNAi, the Golgi number in 2K2N cells fell from 3.5 ± 0.6 to 2.0 ± 0.04 per cell. In 1K1N cells which are non‐dividing or have just entered the cell cycle, cells contain one or 2 Golgi with a mean of 1.8 ± 0.2 per cell. This falls to 1.2 ± 0.1 per cell, and in 2K1N cells from 2.5 ± 0.3 to 1.4 ± 0.1 per cell.

A further analysis of the distribution of the number of Golgi per cell showed that particularly in post‐mitotic (2K2N) cells, the percentage of cells with 4 Golgi decreased significantly (*P* < .001) from 67.0% ± 9.9% to 3.3% ± 1.1% after 8 hours induction of *VSG* RNAi (Figure 4B). Concurrently there was a significant increase in cells with one (1.1 ± 2.0% to 21.2 ± 3.1%, *P* < .01) or 2 Golgi (4.3% ± 3.8% to 47.2% ± 5.1%, *P* < .001). In 1K1N cells, there was a reduction in the percentage of cells with 3 Golgi (11.9% ± 4.8% to 1.9% ± 0.1%, *P* < .05) and a concurrent increase in cells with one Golgi (29.4% ± 7.3% to 67.0% ± 7.0%, *P* < .05) or 2 (57.0 ± 1.8 to 25.7 ± 6.6, *P* < .05). In 2K1N cells, there was a reduction in cells with 3 Golgi (40.3% ± 9.8% to 10.1% ± 1.8%, *P* < .01) and a concomitant increase in the percentage of cells with one Golgi (6.4% ± 4.4% to 60.6% ± 9.4%, *P* < .01). Similar results were obtained after 12 hours induction of *VSG* RNAi. All of these data argue that Golgi number is affected by the amount of secretory cargo present in the cell, and in the presence of an extreme reduction in cargo the number of Golgi per cell is reduced. Here, Golgi body numbers in dividing cells remain similar to Golgi numbers in non‐dividing cells when secretory cargo is severely decreased.

In wild type (WT) BF *T. brucei*, less than 1% of the cells had ERES, but no Golgi. The percentage of cells without Golgi increased after induction of a VSG synthesis block, with this increase becoming statistically significant 12 hours after induction (*P* < .05). In 1K1N cells the increase was from 0.5% ± 0.3% to 4.8% ± 2.0% Golgi per cell, in 2K1N cells from undetectable to 9.2% ± 3.3% and in 2K2N cells from undetectable to 6.6% ± 2.6%. GRASP is one of the first components recruited during assembly of the Golgi in *T. brucei,* 28 and cells without GRASP signal are unlikely to have assembled a new Golgi body. Assembly of Golgi bodies *de novo* in yeast is dependent on COPII export from the ER, which nucleates the formation of a new Golgi body through fusion with vesicles originating from pre‐existing Golgi.^29^ Given the asynchronous nature of cells where *VSG* RNAi has been induced, the small proportion of cells where Golgi were not visible could be daughters of cells with reduced Golgi that did not inherit a Golgi body after cytokinesis.

### 2.3 Distortion of the ER and dilation of Golgi cisternae in the presence of a VSG synthesis block

**Figure 5:**
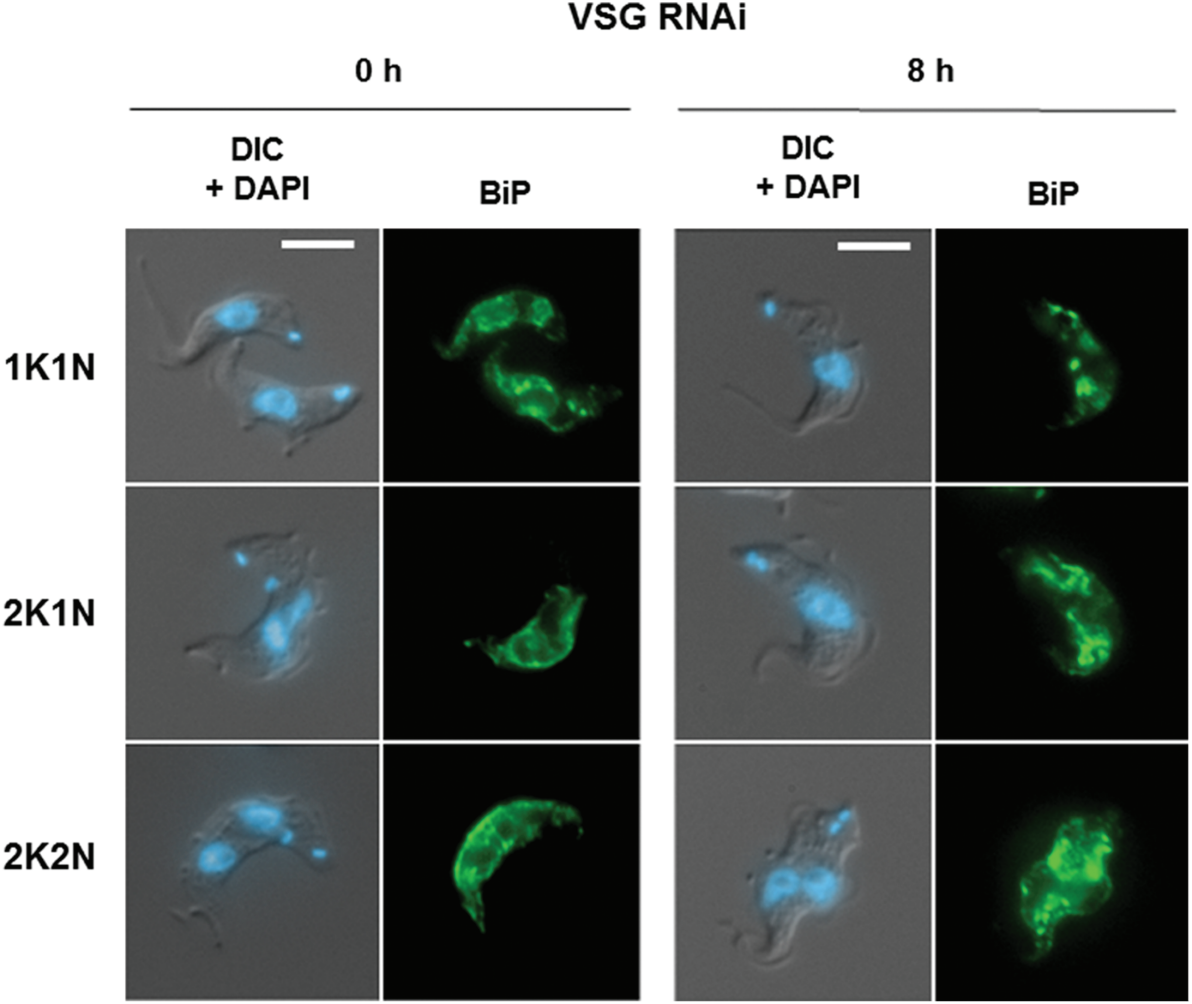
Blocking VSG synthesis results in distortion of the ER. Representative immunofluorescence microscopy images of Trypanosoma brucei VB1.1 cells either before or after the induction of *VSG221* RNAi for 8 hours (h). Trypanosomes are visualised with differential interference contrast (DIC) and with the DNA stained with DAPI, or the ER visualised using an anti‐BiP antibody. Representative cells at different stages of the cell cycle (1K1N, 2K1N and 2K2N) are shown at equivalent exposure times. Scale bar is 5 μM

Staining BF *T. brucei* with antibodies against BiP (binding immunoglobulin protein) allows for the visualisation of the ER, which forms a continuous network throughout the cell.^30^, ^31^ In cells where VSG synthesis had been blocked, the organisation of this ER network was disturbed, and the ER appeared distorted irrespective of cell cycle stage (Figure 5). This could be a consequence of decreased vesicular traffic out of the ER. In other experimental systems, structural distortion of the ER can be a consequence of ER stress, which typically occurs after the accumulation of misfolded protein.^32^, ^33^, ^34^

**Figure 6:**
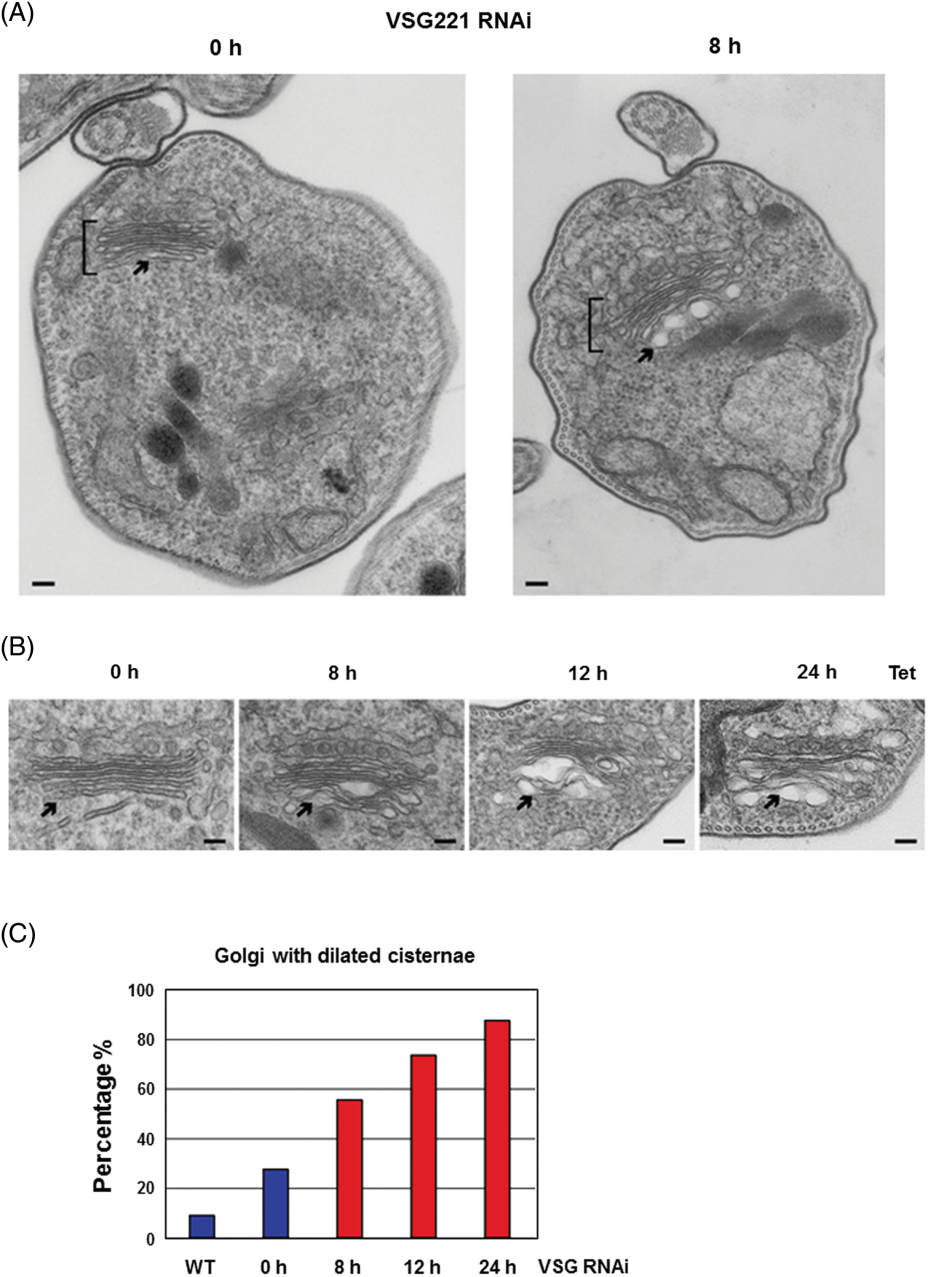
Halting VSG synthesis results in dilation of Golgi cisternae particularly at the Golgi trans‐face. A, Transmission electron microscopy (TEM) analysis of Trypanosoma brucei VB1.1 where *VSG* RNAi has been induced for 0 or 8 hours (h). The Golgi stacks are indicated with brackets, and the Golgi trans‐face with an arrow. The scale bar represents 100 nm. B, Prolonged induction of *VSG* RNAi with tetracycline (Tet) for the time indicated in hours (h) results in progressive dilation of Golgi cisternae, predominantly at the trans‐face (indicated with an arrow). C, Quantitation of the percentage of Golgi with dilated cisternae in T. brucei 427 WT cells or T. brucei VB1.1 where *VSG* RNAi has been induced for 0 or 8 hours. The total number of Golgi counted are *n* = 33 for WT, *n* = 51 for uninduced, *n* = 53 for 8 hours, *n* = 23 for 12 hours and *n* = 24 for 24 hours induction of *VSG* RNAi. Out of focus Golgi were not included in the analyses

The Golgi in BF *T. brucei* appears as a stack of 5 to 6 flattened cisternae (Figure 6A; Figure S3A). However, after the induction of *VSG221* RNAi for 8 hours, gross distension can be observed in the last 2 or 3 cisternae of the trans‐Golgi (Figure 6A,B). The proportion of Golgi stacks with dilated cisternae increases to over 80% in a time dependent manner over a period of 8 to 24 hours (Figure 6C). There was no evidence for a significant increase in cisternae per stack (Figure S3B). VSG constitutes the bulk of secreted protein in the BF trypanosome, so blocking its synthesis would be expected to radically reduce the amount of transitory protein cargo in the Golgi. If secretory vesicles only bud off from the Golgi cisternae in the presence of enough cargo to fill them, this would lead to a reduction in the number of secretory vesicles leaving the Golgi for the plasma membrane. This reduction in exiting vesicles in the face of continuous flow of membrane components into the Golgi cisternae could result in the accumulation of membrane components or unusual lipid to cargo protein ratios in the Golgi. This appears to be what we observe, where membrane accumulation could explain these dramatic distortions of the cisternae.

### 2.4 Vesicular trafficking is relatively unaffected by depletion of the major secretory cargo

**Figure 7:**
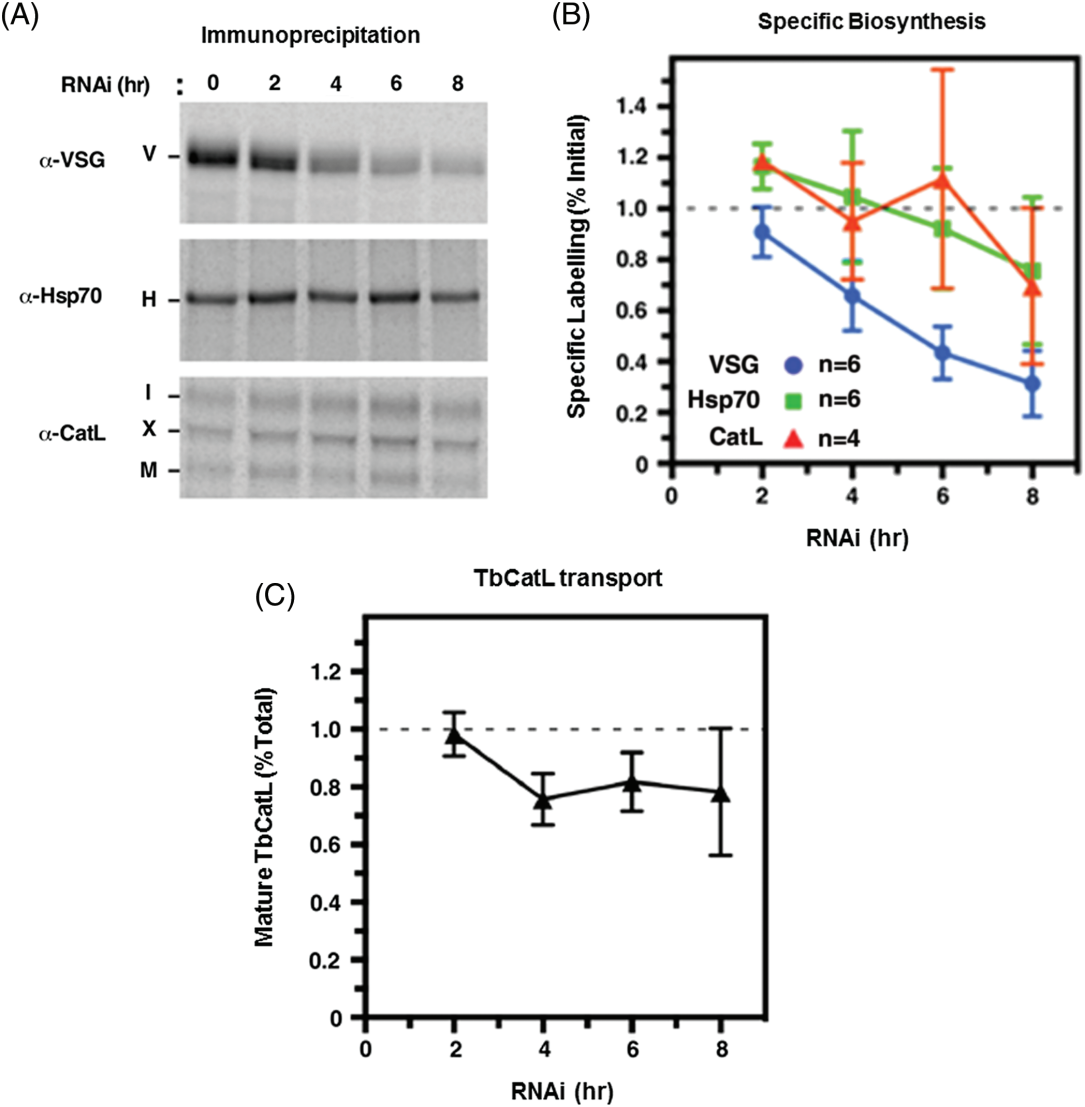
Progression through the secretory pathway is still functional in Trypanosoma brucei where VSG synthesis has been blocked with RNAi. Cultures of T. brucei (2 x 10^5^ cells ml^−1^) had *VSG* RNAi induced for the time indicated in hours (h). Aliquots were removed and subjected to pulse radiolabelling for 15 minutes with [^35^S]Meth/ cysteine followed by specific immunoprecipitation from the cell extracts using antibodies against VSG221, Hsp70 or TbCatL. Immunoprecipitates were fractionated by SDS‐PAGE. A, Representative Phosphorimages are shown of immunoprecipitates (VSG, 0.5 × 10^5^ cell equivalents/ lane and Hsp70 and TbCatL 10^7^ cell equivalents/ lane). The relevant proteins are indicated: VSG221 (V), Hsp70 (H), TbCatL precursors (I and X) and mature TbCatL (M). All immunoprecipitates are from the same radiolabelling experiment. B, Quantification of specific Phosphorimage signals compared with uninduced cells for the indicated number of biological replicates (n), with SD shown with error bars. The CatL signal is the total signal of both the precursor and mature forms (I + X + M). C, Transport of TbCatL from the ER to the lysosome was determined as the ratio of mature TbCatL (M) to total TbCatL (I + X + M) at each time point of induction of *VSG* RNAi normalised to T_0_

We next determined if the changed morphology of the cisternae of the trans‐Golgi was accompanied by a disruption of Golgi function, and investigated secretory traffic in these stalled cells. VSG comprises the vast majority of the total secretory cargo. However, in order to determine if blocking its synthesis and export affects the trafficking of other secretory cargo, we followed the maturation of TbCatL, a soluble lysosomal Cathepsin L orthologue. TbCatL is initially synthesised as a doublet of ER pro‐proteins (I, 53 kDa; X, 50 kDa) that are rapidly processed (*t*_1/2_ 10‐12 minutes) by proteolytic cleavage to the mature enzyme (M, 44 kDa) upon arrival in the lysosome.^35^, ^36^ Appearance of mature TbCatL, therefore, provides a kinetic measure of forward trafficking from the ER. *VSG221* RNAi was induced for an 8 hours window, during which period VSG synthesis was expected to fall dramatically, while synthesis of other proteins would remain relatively unaffected.^21^ Aliquots of cells were removed at 2‐hour intervals and subjected to pulse radiolabelling. This was followed by specific immunoprecipitation of VSG221, cytoplasmic Hsp70 and TbCatL polypeptides from cell extracts (Figure 7B). As expected, after the induction of *VSG221* RNAi, VSG synthesis steeply declined to about 30% normal levels by 8 hours. During this period, synthesis of Hsp70 was only modestly reduced. Similarly, synthesis of TbCatL was only modestly affected. Both Hsp70 and TbCatL synthesis declined to approximately 70% normal levels by 8 hours. More importantly, the fraction of mature TbCatL remained relatively constant. These results indicate that vesicular trafficking from the ER was still ongoing, albeit at a reduced level (down to approximately 80% normal levels) despite the relative emptying of the secretory pathway through the loss of VSG cargo.

### 2.5 Lipid biosynthesis is largely unaffected in cells arrested after induction of *VSG* RNAi

**Figure 8:**
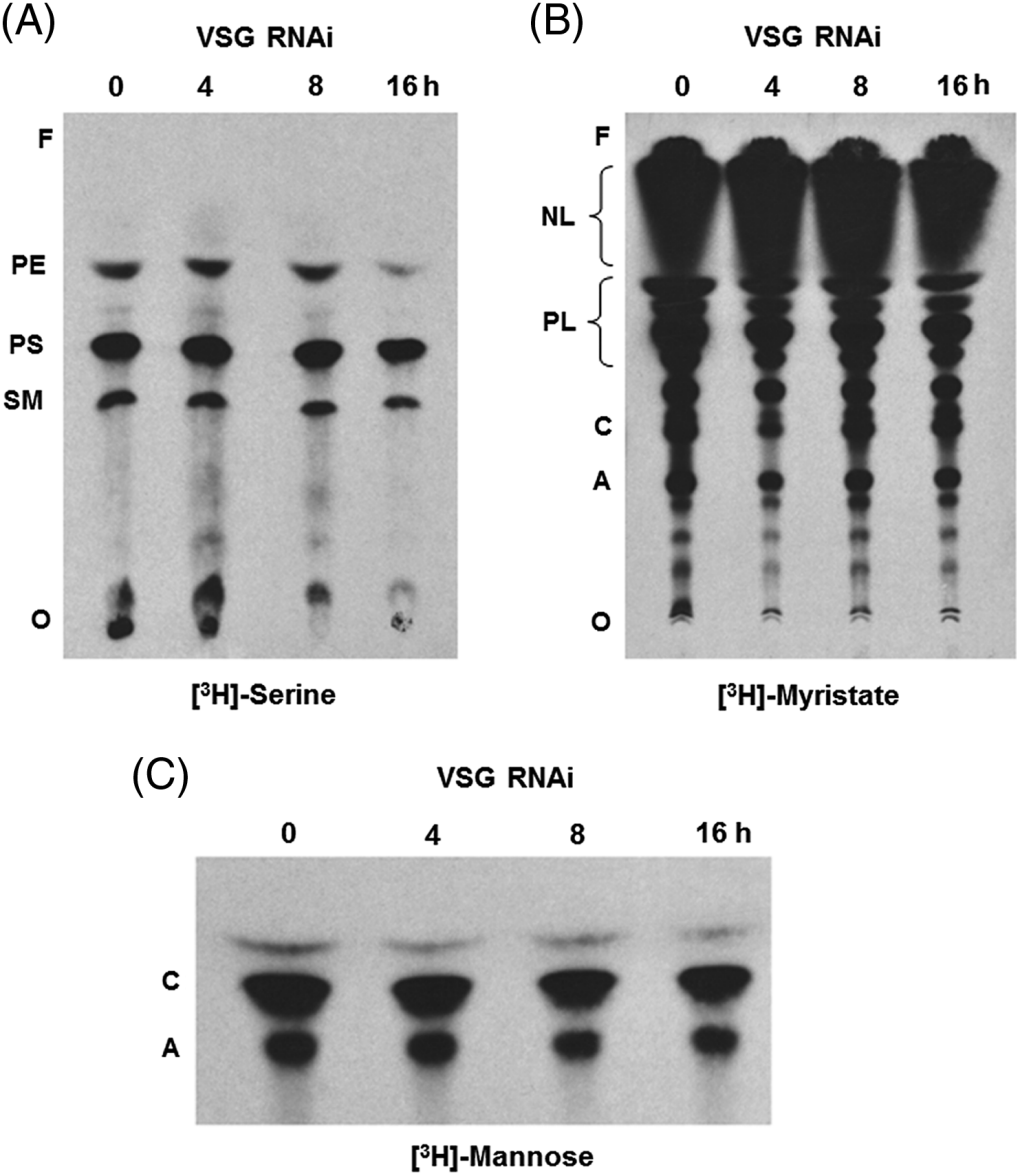
Lipid and GPI‐anchor biosynthesis continues in stalled cells. A, *VSG* RNAi was induced in Trypanosoma brucei VB1.1 for the time indicated in hours (h) prior to metabolic labelling with [^3^H]serine, after which lipids were extracted, desalted, separated by HPTLC and [^3^H]‐labelled lipids detected by fluorography. Radiolabeled PE, PS and SM are indicated as well as the origin (O) and the front (F). B, Cells were labelled and processed as in panel A, but using [^3^H]myristate. NL and PL are indicated with brackets, as well as glycolipid C (C) and glycolipid A (A), and the origin (O) and the front (F). C, Cells were labelled and processed as above, but using [^3^H]‐mannose. Glycolipid C (C) and glycolipid A (A) are indicated on the left

As we saw distortion of the ER and dilation of the trans‐cisternae of the Golgi after the induction of a VSG synthesis block, we also investigated if there was any perturbation of lipid biosynthesis/homeostasis. We have shown previously that there is no reduction of incorporation of [^3^H]‐serine into the lipid fraction in cells when VSG synthesis had been blocked, showing that lipid synthesis was still occurring.^21^ Here we further investigate which lipids were labelled with [^3^H]‐serine. Prior to induction of *VSG* RNAi, 3 lipids were [^3^H]‐labelled as expected: phosphatidyl‐ethanolamine (PE), phosphatidylserine (PS) and sphingomyelin (SM) (Figure 8A, lane 0).^37^ After inducing *VSG* RNAi over a period of 8 hours, no significant differences in the [^3^H]‐serine labelling of the lipids was observed (Figure 8A, lane 4, 8 hours), compared to the uninduced cells (Figure 8A, lane 0 hour). However, there was a noticeable decrease in the amount of PE labelled, and a slight decrease in SM and PS labelling at 16 hours (Figure 8A, lanes 16 hours), which could be related to a reduction in membrane lipid biogenesis due to the overall stalling of the cell‐cycle.

The corresponding [^3^H]‐myristate lipid labelling of uninduced cells (Figure 8B, lane 0 hour) shows various lipid species ranging from neutral lipids (NL) migrating with the front (F), various phospholipids (PL) and mature dimyristylated‐GPI anchor precursors: glycolipids A and C. After induction of VSG RNAi, all of the [^3^H]‐myristate lipid species were still formed, including the GPI anchor glycolipids A and C (compare Figure 8B, lane 0 with lanes 4, 8 and 16 hours *VSG* RNAi). This was confirmed by the quantification of [^3^H]‐myristate taken up and incorporated into the lipid fraction, which is essentially unchanged in cells stalled after blocking VSG synthesis for up to 24 hours (Figure S4A).

However, the [^3^H]‐myristate incorporation into the protein pool does decrease over this period (Figure S4A). As expected, this is primarily due to the reduction in biosynthesis of newly formed VSG protein in the ER, and subsequent GPI‐anchor addition via a transamidase reaction.^38^ Nevertheless, myristate incorporation into protein is not completely abolished after 24 hours induction of *VSG* RNAi, as there are several other less abundant GPI‐anchored proteins such as transferrin receptor that will likely still undergo GPI addition, and direct myristylation of cytosolic proteins will still be ongoing.^39^ In addition, pre‐existing cell surface VSG will still undergo endocytosis, early endosomal sorting, and post‐translational myristate‐exchange prior to being trafficked back to the cell surface.^40^

Uptake and utilisation of [^3^H]mannose was next investigated. Mannose can be incorporated into glycoproteins primarily via N‐glycosylation, but also in GPI‐anchors, both of which are important for the formation of mature VSG. It is, therefore, unsurprising that incorporation of [^3^H]mannose into protein decreased significantly, and in keeping with the decreased rate of VSG synthesis (Figure S4B), as well as decreased protein synthesis in general.^21^ GPI anchor biosynthesis was confirmed to be unaffected after the induction of *VSG* RNAi using the more sensitive [^3^H]mannose labelling, which shows equal amounts of glycolipid A and C formation in both uninduced (Figure 8C, lane 0 hour) and induced cells (Figure 8C, lanes 4, 8 and 16 hours *VSG* RNAi), thus leaving the levels of mannose incorporation into the lipid pool constant (Figure S4B). Unexpectedly, the total rate of uptake of [^3^H]mannose decreased, but down to only ~50% of its original level after 24 hours induction of VSG RNAi. This is some 5‐fold higher than is required for the remaining unchanged GPI anchor biosynthesis. This uptake and possible utilisation of [^3^H]‐mannose by the trypanosome is at present unexplained. Collectively, these data indicate that the biosynthesis of both phospholipids and GPI‐anchors is largely unaffected as the trypanosome tries to maintain lipid homeostasis during the cell‐cycle arrest caused by blocking VSG synthesis.

### 2.6 Lipid levels in arrested cells

As shown earlier, lipid biosynthesis proceeded normally after the induction of a VSG synthesis block induced cell cycle arrest. However, as we had observed abnormalities in the membranes of the ER and Golgi (Figure 6), we next investigated if there were any cumulative changes to particular phospholipid levels. Comparative phospholipid analysis by nano‐ESI‐MS‐MS was employed to analyse the phospholipid composition before and after induction of VSG RNAi for 16 hours. Survey scans in negative ion mode of phospholipid extracts of the cells showed no significant lipid changes (compare Figure 4C, 0 hour vs 16 hours *VSG* RNAi), with the exception of an increase in ethanolamine‐phosphoceramide (EPC) d34:1 at 659 m/z. Spectra for the individual phospholipid classes PG, PE, PS and PI from induced cells remained largely unchanged when compared with the phospholipid composition of uninduced cells (data not shown).

**Figure 9:**
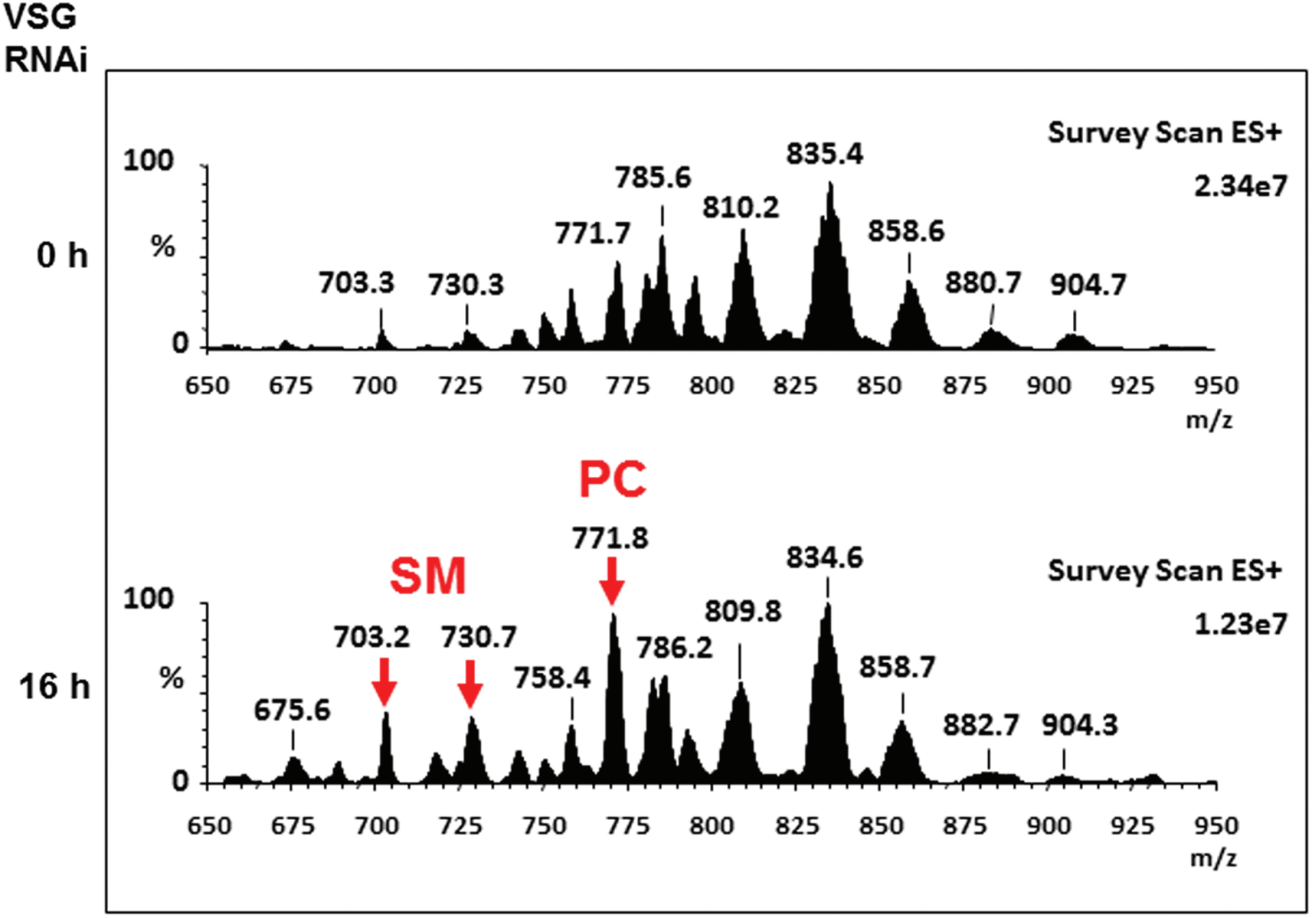
Lipidomic analysis of Trypanosoma brucei in the presence or absence of *VSG* RNAi for 16 hours. Survey ESI‐MS in positive ion mode (600‐1000 *m*/*z*) of lipid extracts from T. brucei VG1.1 that had been cultured in the presence or absence of *VSG* RNAi for 0 or 16 hours (h). The red arrows indicate the species which increase significantly upon induction of *VSG* RNAi, which were confirmed as being choline‐phosphate containing SM or PC species (Figure S3C and Table S1)

In contrast, the positive ion mode survey scans which visualise choline containing phospholipids showed drastic changes in the relative intensities of the phospholipid species in the cells before and after induction of VSG RNAi (Figure 9). These changes included an increase in sphingomyelin (SM) and phosphatidylcholine (PC) species. In order to compare relative potential differences in the choline‐containing phospholipids, precursor *m/z* 184 ion (corresponding to choline‐phosphate fragment ion) spectra were obtained (Figure S5). The SM and PC species were annotated and assigned based upon their [M + H]^+^ and daughter ion masses. The respective percentages of the peaks of the total ion current were calculated, allowing semi‐quantitative comparisons between uninduced and induced cells (Table S1). There was a relatively striking increase in 4 main choline containing species, which increased dramatically after 16 hours induction of *VSG* RNAi. These species were identified as *m/z* 772, alkenylacyl C36:2 (number of FA carbons: their degrees of saturation); *m/z* 744, alkenylacyl C34:2; *m/z* 730, sphingomyelin C18:0; and *m/z* 70, sphingomyelin C16:0.^41^ While the main diacyl‐PC species with the exception of *m/z* 786, diacyl C36:2, show a relative decrease.

This lipidomic analysis suggests that despite lipid biosynthesis in general not being altered significantly during *VSG* RNAi, there is a disproportionate increase in SM levels, suggesting an accumulation, most likely due to a lack of catabolism as the rate of synthesis does not seem to increase (Figure 8A). We, therefore, show here that the cell‐cycle arrest induced by a block in VSG synthesis has very striking characteristics. Cells do not grow, but are metabolically active with no major changes in lipid or GPI‐anchor biosynthesis, but certain phospholipids (ie, SM) accumulate, presumably as a result of not being turned over (catabolised).

## 3 DISCUSSION

In summary, we show that blocking VSG synthesis at the level of its translation produces an equivalent cell cycle arrest to that derived after ablating *VSG* transcript. Blocking synthesis of the major secretory protein of BF African trypanosomes also results in striking changes to both the number and morphology of secretory structures and organelles. There is a reduction in number of both ERES and Golgi bodies. This is compatible with the hypothesis that the maintenance of these structures and organelles is linked to the amount of secretory cargo that passes through them. Induction of a VSG synthesis block also resulted in distorted ER and Golgi morphology, with an apparent increasing accumulation of membrane, particularly in the cisternae at the trans‐face of the Golgi. This could argue that secretory vesicles only bud off from the Golgi if there is sufficient cargo to fill them. However, despite the severe changes in morphology of secretory organelles, the secretory pathway of the stalled cells still appeared to be functional, as transport of endogenous TbCatL to the lysosome was unaffected. Furthermore, there were no observable gross changes in lipid biosynthesis.

Although blocking VSG synthesis triggers a specific pre‐cytokinesis checkpoint, it is unknown what aspect of VSG is “sensed” during trypanosome progression through the cell cycle. We, therefore, investigated here if VSG mRNA depletion, or alternatively a reduction in total amount of VSG protein or protein synthesis was key in triggering this checkpoint. We show that blocking VSG synthesis at the level of its translation, while leaving levels of *VSG* mRNA unaffected, produced an equivalent cell cycle arrest to that induced by the ablation of *VSG* mRNA. This eliminates mRNA as the target being “sensed” in this pathway, and argues that it is the total amount of VSG protein or its synthesis or trafficking to the cell surface that is being monitored to trigger this intriguing cell cycle arrest.

After inducing VSG RNAi for only 8 hours, there was an observed reduction in the number of both ERES and Golgi bodies. There is likely to be an interplay between the amount of cargo and ERES. Overexpression of secretory cargo has been shown to result in more ERES in other experimental systems, as in B cells stimulated to produce large amounts of secreted immunoglobulins, there is an almost 4‐fold increase in the number of ERES.^42^ The ERES are juxtaposed to the Golgi in *T. brucei,* 3, 43 and are duplicated at the same time during *T. brucei* cell division.^12^, ^14^, ^28^ After blocking VSG synthesis, in addition to a reduction in the number of ERES, we also saw a significant decrease in the number of Golgi bodies.

In a model supported by plant experimental systems, it has been proposed that both ERES and Golgi biogenesis is linked to the amount of bulk secretory traffic in the cell.^44^, ^45^ Here, it was proposed that Golgi bodies can be generated directly from ERES, and in this scenario the number of Golgi in the cell is directly affected by the number of ERES on the ER surface. This would allow the cell to dynamically respond to variations in the synthesis of different secretory proteins, allowing it to modify its subcellular secretory machinery subject to requirement. Our data is compatible with this model.

Although during *T. brucei* cell division the new Golgi does not appear to be an exclusive product of the ERES,^12^, ^28^ there could still be feedback between the two subcellular structures. The Golgi is clearly a very dynamic structure, and its structure and function has been argued to rely on the integrity of export from the ER.^46^ Similarly, when mammalian cells were depleted of Golgi bodies using laser nanosurgery, there was a reduction in the number of ERES and reduced ER export.^47^ This argues for an interplay between the two structures. Although Golgi biogenesis in *T. brucei* is likely to involve both *de novo* as well as templated organellar biogenesis, as has been argued to be the case in plants,^12^, ^16^, ^48^ our results suggest that the total amount of secretory cargo also plays an additional modulatory role on Golgi number, which has also been postulated to be the case in plants.^44^ Our data are, therefore, compatible with a model whereby ERES and Golgi biogenesis and maintenance are stimulated by the amount of secretory cargo, which could give us additional insight into Golgi biology in *T. brucei.* 15

When the secretory pathway is emptied of VSG through the induction of a synthesis block, the stalled cells had Golgi with an unchanged number of cisternae per stack. This is compatible with a proposed “stable cisternae” model, whereby each cisterna contains a stable complement of Golgi enzymes.^49^ However, the stalled cells showed distortion of both the ER and the Golgi. The size of the ER can be controlled subject to requirement in different types of eukaryotic cells,^50^ and both the ER and Golgi can expand enormously in B cells producing vast amounts of secreted immunoglobulins.^42^, ^51^ Distortion of the ER lumen can also be produced by ER stress.^32^, ^33^, ^34^ This can occur when the unfolded protein response (UPR) is triggered, and the ER is clogged with either misfolded or overexpressed protein.^52^, ^53^, ^54^ For example, in mice where a genetic perturbation results in misfolded major histocompatibility complex Class I accumulating in the ER, the ER‐Golgi compartment becomes an expanded and distorted tubular network.^55^ Similarly, disruption of disulphide bond formation of mouse proinsulin leads to a 2.9‐fold increase in dilated ER cisternae and a 4.5 to 5.8‐fold increase in Golgi associated and pre‐Golgi intermediate structures.^56^

We do not know if the distorted ER in our arrested cells is a direct response to the drastic reduction in the amount of secretory cargo trafficking through this organelle, or the consequence of a novel ER stress response resulting from the relative emptying of its predominant cargo. ER stress appears to be sensed differently in *T. brucei* compared with other eukaryotes. In BF *T. brucei* chemical induction of ER stress, or disruption of the ER translocon by RNAi does not result in induction of the expected response to ER stress, which is the UPR.^57^, ^58^ In procyclic form *T. brucei* persistent ER stress leads to a spliced leader RNA silencing pathway (SLS) and programmed cell death.^59^ We have no evidence that this occurs after blocking VSG synthesis in BF *T. brucei.* 21

After blocking VSG synthesis we see grossly distended cisternae in the Golgi bodies, and particularly at the trans‐face. We think that it is most likely that the drastic reduction in the amount of secretory cargo passing through this compartment is directly leading to a reduction in the number of secretory vesicles leaving the trans‐cisternae, as they do not bud off in the absence of VSG cargo to fill them. This could result in membrane accumulation in the distal cisternae. In *T. brucei* budding secretory vesicles from the Golgi are targeted to the flagellar pocket, the only region of the plasma membrane where endocytosis and exocytosis take place.^60^ In *T. brucei*, a restriction preventing empty secretory vesicles from docking with the flagellar pocket would be advantageous, as this would at least temporarily prevent what would otherwise be catastrophic dilution of the protective VSG coat in the presence of inadequate amounts of VSG.

Surprisingly, despite the fact that the stalled cells do not grow in cell volume,^20^ lipid biosynthesis remained relatively unchanged, suggesting that the cells are actively trying to maintain lipid homeostasis, although there was an increase in levels of sphingomyelin. In addition, despite the fact that blocking VSG synthesis removes the main acceptor for GPI anchor addition, GPI anchor biosynthesis also remained unaltered. In the case of GPI‐anchor biosynthesis this may be unsurprising, as there is normally a greater than 10‐fold excess of mature GPI‐anchor formation over what is required for attachment to newly synthesised VSG.^24^, ^25^ The fact that lipid synthesis was largely unaffected in the arrested cells indicates the dynamic nature of lipid homeostasis, where there is a constant turnover of lipids as they are synthesised and degraded (Smith unpublished results). However, a striking feature is the increase in sphingomyelin in the arrested cells.

Mammalian cells have three sphingolipid synthases,^61^, ^62^ one of which is Golgi located and is the primary source of sphingomyelin for inclusion in secretory vesicles departing for the cell surface. This sphingomyelin positively impacts post‐Golgi trafficking and sorting of GPI‐anchored molecules.^63^, ^64^ Trypanosomes have four sphingolipid synthase genes, two of which actively synthesise sphingomyelin in BF parasites (SLS3 and SLS4), and one of which (SLS4) is localised to the Golgi.^65^ However, inhibition of sphingolipid synthesis in trypanosomes does not apparently impact forward trafficking of GPI anchored cargo, either at the level of ER exit or in post‐Golgi compartments,^65^, ^66^ although a conditional knockout of the ER neutral sphingomyelinase had a detrimental effect of post‐Golgi sorting of VSG.^63^ Whatever the reason for the elevated sphingomyelin level, it seems likely that this may contribute to the observed enlargement of the distal cisternae of the Golgi.

In conclusion, we show that blocking synthesis of the major secretory protein of *T. brucei* results in dramatic changes to both the number and morphology of secretory pathway structures and organelles. The reduced number of ERES and Golgi could be explained by a model where the amount of cargo directly impacts on organelle homeostasis, and whereby secretory organelles are created and maintained subject to demand. Similarly, reduced forward trafficking of secretory vesicles in the stalled cells could explain the observed distortions in the secretory organelles. Particularly in the Golgi, reduced budding of secretory vesicles could explain the massive membrane accumulation at the trans‐face that we observe. These data, therefore, highlight the interconnection between secretory cargo and organelle biogenesis and maintenance, which is relevant to understanding these pathways in other eukaryotes in addition to African trypanosomes.

## 4 MATERIALS AND METHODS

### 4.1 Trypanosome strains, culturing and manipulation

BF *Trypanosoma brucei brucei* 427 was used for all experiments. Cells were cultured in HMI‐9 medium with 15% fetal calf serum, but for some experiments requiring tetracycline free conditions, Tet‐system approved fetal calf serum (Clontech) was used. *T. brucei* HNI (221+) with a hygromycin resistance gene selecting for the *VSG221* expression site was used for all Morpholino experiments.^22^ In *T. brucei* 221VB cells *VSG221* RNAi can be induced with tetracycline.^20^ *T. brucei* SL221RNAi cells were generated by transfecting *T. brucei* SM221pur^67^ with the pLEW100.v5x.PEX11.VSG221RNAi construct (stem‐loop RNAi construct derived from^36^). One allele of TbSec24.1 (Tb927.3.1210) was endogenously tagged with Ty1 at the C‐terminus in *T. brucei* SL221RNAi cells using the construct from,^24^ generating the *T. brucei* SL221 TbSec24.1::Ty1 cell line. One allele of TbSec23.2 (Tb927.10.7740) was subsequently tagged in *T. brucei* SL221 TbSec24.1::Ty1 cells using the tagging construct derived from,^24^ but with the neomycin resistance cassette swapped with that of a blasticidin resistance gene. *T. brucei* VB1.1 and *T. brucei* VG1.1 are described in.^20^

### 4.2 Morpholino experiments

Morpholino antisense oligonucleotides (Gene Tools, LLC) were resuspended in 500 μL of cytomix (10 mM K_2_HPO_4_/KH_2_PO_4_ pH 7.6; 2 mM EGTA; 120 mM KCl; 150 μM CaCl_2_; 25 mM HEPES; 5 mM MgCl_2_; 0.5% [w/v] glucose; 1 mM hypoxanthine; 100 μg/mL BSA. pH 7.6). The anti‐*VSG221* (both WT and mismatched) Morpholino oligos were used at an end concentration of 80 μM in the cuvette and the anti‐tubulin (both WT and mismatched) Morpholino oligos were used at an end concentration of 20 μM. Morpholino oligonucleotides were combined with log phase *T. brucei* BSF HNI (221+) cells (2 x 10^7^) and electroporated on a BTX Electro Square Porator, ECM 830 (VWR) at 1500 V.

### 4.3 Northern blot analysis

For Northern blot analyses total isolated RNA (1‐1.5 μg per sample) was mixed with 10 mM MOPS, pH 7.0; 1 mM NaOAc; 0.5 mM EDTA 50% (v/v) formamide; 17.5% (v/v) formaldehyde; 60 μg/mL^−1^ ethidium bromide and denatured for 15 minutes at 65°C. The RNA was resolved on 1.5% formaldehyde‐agarose gel in MOPS buffer. RNA was transferred onto Hybond‐XL membrane (GE Healthcare) using standard protocols. Blots were hybridised with random primed probes that had been radiolabeled with [^32^P]‐dCTP using Amersham Ready‐To‐Go DNA labelling Beads (‐dCTP) (GE Healthcare) following the instructions of the manufacturer. An 803‐bp DNA probe targeting *VSG221* was PCR amplified from *T. brucei* 90‐13 genomic DNA using primers from.^20^ Membranes were washed to an end stringency of 0.1 × SSC at 65°C. The blots were imaged with a Personal Molecular Imager FX (Bio‐Rad).

### 4.4 Microscopy analysis

For immunofluorescence microscopy experiments, cells were washed twice in cold PSG buffer and fixed with 2% paraformaldehyde at room temperature for 15 minutes. Cells were washed twice in PBS buffer and allowed to settle on ColorFrost Plus microscopy slides (Shandon) for 30 minutes in a humidity chamber. For the experiments to quantitate ERES and Golgi, cells were probed with rabbit polyclonal anti‐GRASP primary antibody (gift from Graham Warren) and mouse BB2 (anti‐Ty, gift from Keith Gull) and the appropriate secondary antibodies. For co‐localisation of TbSec23.2::HA and TbSec24.1::Ty1, cells were probed with BB2 and a rabbit anti‐HA antibody (ab9110, Abcam) followed by the appropriate secondary antibodies. Slides were mounted in Vectashield with DAPI (Vector Laboratories). Microscopy analyses were performed on either an Axioplan 2 (Zeiss), an Axio Imager M1, or an Axio Imager M2 (Zeiss), and images were captured with either a Cool Snap HQ (Roper Scientific) camera an AxioCam MRc (Zeiss) camera or an Orca R2 camera (Hamamatsu). Post‐acquisition analyses were carried out with ImageJ (National Institutes of Health).

The *T. brucei* examined by thin section transmission electron microscopy (TEM) were processed as described previously,^20^ and examined in a FEI Tecnai 12 transmission electron microscope.

### 4.5 Trypanosoma brucei metabolic labelling and lipid and protein analysis

Pulse‐chase radiolabelling of log‐phase cultured BF *T. brucei* with [^35^S]methionine/ cysteine [Perkin Elmer, Waltham, Massachusetts], and subsequent immunoprecipitation of labelled polypeptides was performed as described previously.^35^, ^36^ Briefly, cells were harvested, washed and transferred to labelling media at 10^7^ mL^−1^. After a 15 minute pre‐incubation at 37°C, radiolabel was added to 200 μCi mL^−1^ and incubation was continued for an additional 15 minutes. Cells were washed and lysed in RIPA buffer (50 mM Tris‐HCl, pH 8.0, 100 mM NaCl, 1% NP40, 0.5% deoxycholate, 0.1% SDS) at 10^7^ cell equivalents. All antibodies were pre‐coupled to Protein A Sepharose. Immunoprecipitates were fractionated by 12% SDS‐PAGE, and gels were analysed by Phosphorimaging using a Molecular Dynamics Typhoon FLA 9000 system with native ImageQuant software (GE Healthcare).

For the experiments involving lipid or protein analysis, mid‐logarithmic WT BF *T. brucei* or *T. brucei* VG1.1 cells^20^ where *VSG221* RNAi had been induced with tetracycline (1 μg/mL) were centrifuged (800 g for 10 minutes) and washed in minimal essential media (fatty‐acid, glucose or serine free), before suspension in the same media at 1 × 10^7^ cells mL^−1^. Total protein synthesis was inhibited in WT cells by pre‐incubating for 10 minutes at 37°C with cycloheximide (60 μg/mL). Cells were labelled for 1 hour at 37°C with 50 μCi mL^−1^ of [^3^H]serine (20 Ci mmol^−1^, ARC), D‐[2‐^3^H]mannose (14 Ci mmol^−1^, ARC), [^3^H]myristate (47 Ci mmol^−1^, ARC), or 5 μCi mL^−1^ for 30 minutes in a shaking water bath. The cells were collected by centrifugation and washed briefly in TDB buffer (25 mM KCl, 400 mM NaCl, 5 mM MgSO_4_, 100 mM Na_2_HPO_4_/ NaH_2_PO_4_, 100 mM glucose) prior to samples taken for either lipid or protein analysis as previously described.^68^

Lipids were extracted with organic solvents, dried and partitioned between butanol/ water. The desalted lipids (2 × 10^6^ cell equivalents per lane) were separated by HPTLC using silica 60 HPTLC plates and a chloroform:methanol:water (10:10:3 v/v) solvent system. Radiolabeled lipids were detected by fluorography at −70°C, after spraying with En^3^hance and using Kodak XAR‐5 film with an intensifying screen. GPI intermediates were identified using various enzyme and chemical digests as previously described.^69^

For experiments involving radiolabel incorporation into total, protein and lipid fractions *T. brucei* VG1.1 had *VSG221* RNAi induced with tetracycline for varying times, and then the cells were metabolically labelled with [^3^H]serine, [^3^H]mannose or [^3^H]myristate for 1 hour as described above. Cells were split into equal volumes and processed to quantitate radiolabel incorporation into protein by TCA precipitation or lipid by organic extraction or whole cells. Values are the mean with SDs of 3 separate labelling experiments, the values at time 0 are normalised to 100%. The deviation of total incorporation between different experiments was no greater than 10%. For the lipidomic analyses, lipids were extracted and analysed in mid‐log cells in the presence or absence of *VSG221* RNAi for 16 hours as previously described.^70^

## Supporting information


**Editorial Process**

**Appendix S1** Supporting Information
**Figure S1** Morpholino anti‐sense oligonucleotides as a strategy to block VSG synthesis. A, Anti‐sense Morpholino oligonucleotides were designed to bind the area, including and downstream of the start codon (indicated with a box) of *VSG221* or α‐tubulin mRNA. Oligonucleotides either targeted the WT sequence, or had mismatched nucleotides incorporated (indicated in red), which would disrupt oligonucleotide binding. B, Transfection of mismatched α‐tubulin or *VSG221* Morpholinos does not result in phenotypic changes compared with untransfected cells, or Trypanosoma brucei VB1.1 where *VSG221* RNAi has not been induced. Microscopy analysis is shown of cells 12 hours after Morpholino transfection or before induction of *VSG* RNAi visualised using differential interference contrast (DIC) or the DNA stain DAPI. The scale bar indicates 5 μM
**Figure S2** TbSec24.1::Ty1 co‐localises with TbSec23.2::HA in the ERES. A, Immunofluorescence microscopy images showing overlap of TbSec23.2::HA (green) with that of TbSec24::Ty1 (red). Cells were imaged using Differential Interference Contrast (DIC) with DAPI staining of DNA is shown in blue. Scale bar represents 5 μM. B, The signal intensity of TbSec23.2::HA and TbSec24.1::Ty1 along a linear line drawn through the centre of both signals (left panel) was measured (right panel). The displacement of both signals was defined by the sum in the distance of the red and the green signal at the start and end of the peaks. The mean displacement between the 2 signals was 0.03 ± 0.3 μM (*n* = 25 cells that were 1K1N, error in SD). A representative trace is shown
**Figure S3** The total number of cisternae per Golgi stack does not change significantly in cells where VSG synthesis has been blocked for 24 hours (h). A, Schematic showing a transmission electron microscopy (TEM) image of BSF Trypanosoma brucei where the relevant subcellular structures are indicated, with the endoplasmic reticulum (ER) indicated in yellow, the Golgi cisternae in red and vesicles in purple. The different Golgi cisternae are numbered, with the *cis* and trans‐face of the Golgi indicated next to the bracket. B, Quantitation of the number of Golgi cisternae observed in the TEM images. The total number of Golgi counted are *n* = 51 for uninduced and *n* = 24 for 24 hours induction of *VSG221* RNAi. These are the same Golgi that were shown in Figure 6(C). A *χ* ^2^ test shows no significant difference in the number of cisternae (*P* = .1490)
**Figure S4** Quantitation of Trypanosoma brucei metabolic labelling experiments whereby the incorporation of radioactive labelled precursors into whole cells (uptake), total protein or lipids was followed after blocking VSG synthesis for various periods. A, *VSG221* RNAi was induced in T. brucei VG1.1 for the time indicated in hours prior to labelling with [^3^H]myristate. Replicate aliquots of the labelled cells were processed, and incorporation of radiolabel into either the whole cell, total protein or lipid fractions was determined. The values show the means and SDs (indicated with error bars) of 3 separate labelling experiments, whereby the values at time 0 are normalised to 100%. B, As above, but the cells were labelled with [^3^H]‐mannose. C, Lipidomic analysis of T. brucei in the presence or absence of a VSG synthesis block. Survey ESI‐MS in negative ion ode (600‐1000 *m*/*z*) of lipid extracts from T. brucei VG1.1 where *VSG* RNAi had been induced for 0 or 16 hours (h). The red arrow indicates the EPC (d34:1) species which increases significantly after induction of *VSG* RNAi
**Figure S5** Parent‐ion scanning of the collision induced fragment for choline‐phosphate (*m*/*z* 184) by positive ion ESI‐MS‐MS showing phosphatidylcholine (PC) and sphingomyelin (SM) phospholipids of lipid extracts from Trypanosoma brucei. VG1.1 cells with *VSG* RNAi induced for the time indicated in hours (h). The red arrows indicate the species which increase significantly upon induction of *VSG* RNAi. The predominant molecular species have been annotated and quantified by their semi‐quantitative percentage (%) (see Table S1)
**Table S1** Lipid composition of *Trypanosoma brucei* VG1.1 cells in the presence or absence of the induction of VSG RNAi for 24 hours

## References

[1] Cross GA . Identification, purification and properties of clone‐specific glycoprotein antigens constituting the surface coat of Trypanosoma brucei. Parasitology. 1975;71(3):393‐417. 645 10.1017/s003118200004717x

[2] Buscher P , Cecchi G , Jamonneau V , Priotto G . Human African trypanosomiasis. Lancet. 2017;390:2397‐2409. 28673422 10.1016/S0140-6736(17)31510-6

[3] Silverman JS , Form BJD . Function in the trypanosomal secretory pathway. Curr Opin Microbiol. 2012;15(4):463‐468. 22445359 10.1016/j.mib.2012.03.002 PMC3393773

[4] Schwede A , Macleod OJ , MacGregor P , Carrington M . How does the VSG coat of bloodstream form African trypanosomes interact with external proteins? PLoS Pathog. 2015;11(12):e1005259. 10.1371/journal.ppat.1005259 PMC4697842 26719972

[5] Bulow R , Overath P , Davoust J . Rapid lateral diffusion of the variant surface glycoprotein in the coat of Trypanosoma brucei. Biochemistry. 1988;27(7):2384‐2388. 3382629 10.1021/bi00407a020

[6] Hartel AJ , Glogger M , Guigas G , et al. The molecular size of the extra‐membrane domain influences the diffusion of the GPI‐anchored VSG on the trypanosome plasma membrane. Sci Rep. 2015;5:10394. 10.1038/srep10394 PMC5387117 26065579

[7] Gomez‐Navarro N , Miller E . Protein sorting at the ER‐Golgi interface. J Cell Biol. 2016;215(6):769‐778. 27903609 10.1083/jcb.201610031 PMC5166505

[8] Hughes L , Borrett S , Towers K , Starborg T , Vaughan S . Patterns of organelle ontogeny through a cell cycle revealed by whole‐cell reconstructions using 3D electron microscopy. J Cell Sci. 2017;130(3):637‐647. 28049718 10.1242/jcs.198887

[9] Tang D , Wang Y . Cell cycle regulation of Golgi membrane dynamics. Trends Cell Biol. 2013;23(6):296‐304. 23453991 10.1016/j.tcb.2013.01.008 PMC3665639

[10] Dupree P , Sherrier DJ . The plant Golgi apparatus. Biochim Biophys Acta. 1998;1404(1–2):259‐270. 9714825 10.1016/s0167-4889(98)00061-5

[11] Papanikou E , Glick BS . The yeast Golgi apparatus: insights and mysteries. FEBS Lett. 2009;583(23):3746‐3751. 19879270 10.1016/j.febslet.2009.10.072 PMC2788027

[12] He CY , Ho HH , Malsam J , et al. Golgi duplication in Trypanosoma brucei. J Cell Biol. 2004;165(3):313‐321. 15138289 10.1083/jcb.200311076 PMC2172185

[13] Clayton CE , Mowatt MR . The procyclic acidic repetitive proteins of Trypanosoma brucei. Purification and post‐translational modification. J Biol Chem. 1989;264(25):15088‐15093. 2475493

[14] Bangs JD . Replication of the ERES:Golgi junction in bloodstream‐form African trypanosomes. Mol Microbiol. 2011;82(6):1433‐1443. 22026408 10.1111/j.1365-2958.2011.07900.x PMC3237776

[15] Lowe M , Barr FA . Inheritance and biogenesis of organelles in the secretory pathway. Nat Rev Mol Cell Biol. 2007;8(6):429‐439. 17505521 10.1038/nrm2179

[16] Yavuz S , Warren G . A role for Sar1 and ARF1 GTPases during Golgi biogenesis in the protozoan parasite Trypanosoma brucei. Mol Biol Cell. 2017;28(13):1782‐1791. 28495798 10.1091/mbc.E17-03-0151 PMC5491186

[17] Morin‐Ganet MN , Rambourg A , Deitz SB , Franzusoff A , Kepes F . Morphogenesis and dynamics of the yeast Golgi apparatus. Traffic. 2000;1(1):56‐68. 11208060 10.1034/j.1600-0854.2000.010109.x

[18] Engstler M , Pfohl T , Herminghaus S , et al. Hydrodynamic flow‐mediated protein sorting on the cell surface of trypanosomes. Cell. 2007;131(3):505‐515. 17981118 10.1016/j.cell.2007.08.046

[19] Cheung JL , Wand NV , Ooi CP , Ridewood S , Wheeler RJ , Rudenko G . Blocking synthesis of the variant surface glycoprotein coat in trypanosoma brucei leads to an increase in macrophage phagocytosis due to reduced clearance of surface coat antibodies. PLoS Pathog. 2016;12(11):e1006023. 27893860 10.1371/journal.ppat.1006023 PMC5125712

[20] Sheader K , Vaughan S , Minchin J , Hughes K , Gull K , Rudenko G . Variant surface glycoprotein RNA interference triggers a precytokinesis cell cycle arrest in African trypanosomes. Proc Natl Acad Sci USA. 2005;102(24):8716‐8721. 15937117 10.1073/pnas.0501886102 PMC1150830

[21] Smith TK , Vasileva N , Gluenz E , et al. Blocking variant surface glycoprotein synthesis in Trypanosoma brucei triggers a general arrest in translation initiation. PLoS One. 2009;4(10):e7532. 10.1371/journal.pone.0007532 PMC2762041 19855834

[22] Rudenko G , Chaves I , Dirks‐Mulder A , Borst P . Selection for activation of a new variant surface glycoprotein gene expression site in *Trypanosoma brucei* can result in deletion of the old one. Mol Biochem Parasitol. 1998;95:97‐109. 9763292 10.1016/s0166-6851(98)00099-1

[23] Ngo H , Tschudi C , Gull K , Ullu E . Double‐stranded RNA induces mRNA degradation in Trypanosoma brucei. Proc Natl Acad Sci USA. 1998;95(25):14687‐14692. 9843950 10.1073/pnas.95.25.14687 PMC24510

[24] Sevova ES , Bangs JD . Streamlined architecture and glycosylphosphatidylinositol‐dependent trafficking in the early secretory pathway of African trypanosomes. Mol Biol Cell. 2009;20(22):4739‐4750. 19759175 10.1091/mbc.E09-07-0542 PMC2777104

[25] Duszenko M , Ivanov IE , Ferguson MA , Plesken H , Cross GA . Intracellular transport of a variant surface glycoprotein in Trypanosoma brucei. J Cell Biol. 1988;106(1):77‐86. 3339091 10.1083/jcb.106.1.77 PMC2114957

[26] Bangs JD , Andrews NW , Hart GW , Englund PT . Posttranslational modification and intracellular transport of a trypanosome variant surface glycoprotein. J Cell Biol. 1986;103(1):255‐263. 3722267 10.1083/jcb.103.1.255 PMC2113794

[27] Sherwin T , Gull K . The cell division cycle of Trypanosoma brucei brucei: timing of event markers and cytoskeletal modulations. Philos Trans R Soc Lond B Biol Sci. 1989;323(1218):573‐588. 2568647 10.1098/rstb.1989.0037

[28] Ho HH , He CY , de Graffenried CL , Murrells LJ , Warren G . Ordered assembly of the duplicating Golgi in Trypanosoma brucei. Proc Natl Acad Sci USA. 2006;103(20):7676‐7681. 16672362 10.1073/pnas.0602595103 PMC1472504

[29] Reinke CA , Kozik P , Glick BS . Golgi inheritance in small buds of *Saccharomyces cerevisiae* is linked to endoplasmic reticulum inheritance. Proc Natl Acad Sci U S A. 2004;101(52):18018‐18023. 15596717 10.1073/pnas.0408256102 PMC539800

[30] Bangs JD , Uyetake L , Brickman MJ , Balber AE , Boothroyd JC . Molecular cloning and cellular localization of a BiP homologue in Trypanosoma brucei. Divergent ER retention signals in a lower eukaryote. J Cell Sci. 1993;105(Pt 4):1101‐1113. 8227199 10.1242/jcs.105.4.1101

[31] Alexander DL , Schwartz KJ , Balber AE , Bangs JD . Developmentally regulated trafficking of the lysosomal membrane protein p67 in Trypanosoma brucei. J Cell Sci. 2002;115(Pt 16):3253‐3263. 12140257 10.1242/jcs.115.16.3253

[32] Anding AL , Baehrecke EH . Cleaning house: selective autophagy of organelles. Dev Cell. 2017;41(1):10‐22. 28399394 10.1016/j.devcel.2017.02.016 PMC5395098

[33] Hotamisligil GS , Davis RJ . Cell signaling and stress responses. Cold Spring Harb Perspect Biol. 2016;8(10):1‐20. https://doi.org/10.1101/cshperspect.a006072. 10.1101/cshperspect.a006072 PMC5046695 27698029

[34] Wang M , Kaufman RJ . Protein misfolding in the endoplasmic reticulum as a conduit to human disease. Nature. 2016;529(7586):326‐335. 26791723 10.1038/nature17041

[35] Silverman JS , Muratore KA , Bangs JD . Characterization of the late endosomal ESCRT machinery in Trypanosoma brucei. Traffic. 2013;14(10):1078‐1090. 23905922 10.1111/tra.12094 PMC3806108

[36] Silverman JS , Schwartz KJ , Hajduk SL , Bangs JD . Late endosomal Rab7 regulates lysosomal trafficking of endocytic but not biosynthetic cargo in Trypanosoma brucei. Mol Microbiol. 2011;82(3):664‐678. 21923766 10.1111/j.1365-2958.2011.07842.x PMC4324464

[37] Farine L , Jelk J , Choi JY , et al. Phosphatidylserine synthase 2 and phosphatidylserine decarboxylase are essential for aminophospholipid synthesis in Trypanosoma brucei. Mol Microbiol. 2017;104(3):412‐427. 28142188 10.1111/mmi.13637 PMC5413845

[38] Kinoshita T , Inoue N . Dissecting and manipulating the pathway for glycosylphos‐phatidylinositol‐anchor biosynthesis. Curr Opin Chem Biol. 2000;4(6):632‐638. 11102867 10.1016/s1367-5931(00)00151-4

[39] Goldston AM , Sharma AI , Paul KS , Engman DM . Acylation in trypanosomatids: an essential process and potential drug target. Trends Parasitol. 2014;30(7):350‐360. 24954795 10.1016/j.pt.2014.05.003 PMC4190163

[40] Paul KS , Jiang D , Morita YS , Englund PT . Fatty acid synthesis in African trypanosomes: a solution to the myristate mystery. Trends Parasitol. 2001;17(8):381‐387. 11685899 10.1016/s1471-4922(01)01984-5

[41] Richmond GS , Gibellini F , Young SA , et al. Lipidomic analysis of bloodstream and procyclic form Trypanosoma brucei. Parasitology. 2010;137(9):1357‐1392. 20602846 10.1017/S0031182010000715 PMC3744936

[42] Kirk SJ , Cliff JM , Thomas JA , Ward TH . Biogenesis of secretory organelles during B cell differentiation. J Leukoc Biol. 2010;87(2):245‐255. 19889725 10.1189/jlb.1208774

[43] Yelinek JT , He CY , Warren G . Ultrastructural study of Golgi duplication in Trypanosoma brucei. Traffic. 2009;10(3):300‐306. 19207482 10.1111/j.1600-0854.2008.00873.x

[44] Hawes C , Schoberer J , Hummel E , Osterrieder A . Biogenesis of the plant Golgi apparatus. Biochem Soc Trans. 2010;38(3):761‐767. 20491662 10.1042/BST0380761

[45] daSilva LL , Snapp EL , Denecke J , Lippincott‐Schwartz J , Hawes C , Brandizzi F . Endoplasmic reticulum export sites and Golgi bodies behave as single mobile secretory units in plant cells. Plant Cell. 2004;16(7):1753‐1771. 15208385 10.1105/tpc.022673 PMC514159

[46] Ward TH , Polishchuk RS , Caplan S , Hirschberg K , Lippincott‐Schwartz J . Maintenance of Golgi structure and function depends on the integrity of ER export. J Cell Biol. 2001;155(4):557‐570. 11706049 10.1083/jcb.200107045 PMC2198855

[47] Ronchi P , Tischer C , Acehan D , Pepperkok R . Positive feedback between Golgi membranes, microtubules and ER exit sites directs de novo biogenesis of the Golgi. J Cell Sci. 2014;127(Pt 21):4620‐4633. 25189616 10.1242/jcs.150474

[48] Abiodun MO , Matsuoka K . Evidence that proliferation of golgi apparatus depends on both de novo generation from the endoplasmic reticulum and formation from pre‐existing stacks during the growth of tobacco BY‐2 cells. Plant Cell Physiol. 2013;54(4):541‐554. 23361898 10.1093/pcp/pct014

[49] Warren G . Transport through the Golgi in Trypanosoma brucei. Histochem Cell Biol. 2013;140(3):235‐238. 23765165 10.1007/s00418-013-1112-y

[50] Schuck S . On keeping the right ER size. Nat Cell Biol. 2016;18(11):1118‐1119. 27784902 10.1038/ncb3430

[51] Federovitch CM , Ron D , Hampton RY . The dynamic ER: experimental approaches and current questions. Curr Opin Cell Biol. 2005;17(4):409‐414. 15975777 10.1016/j.ceb.2005.06.010

[52] Lee J , Ozcan U . Unfolded protein response signaling and metabolic diseases. J Biol Chem. 2014;289(3):1203‐1211. 24324257 10.1074/jbc.R113.534743 PMC3894306

[53] McCaffrey K , Braakman I . Protein quality control at the endoplasmic reticulum. Essays Biochem. 2016;60(2):227‐235. 27744338 10.1042/EBC20160003

[54] Kaufman RJ . Orchestrating the unfolded protein response in health and disease. J Clin Invest. 2002;110(10):1389‐1398. 12438434 10.1172/JCI16886 PMC151822

[55] Raposo G , van Santen HM , Leijendekker R , Geuze HJ , Ploegh HL . Misfolded major histocompatibility complex class I molecules accumulate in an expanded ER‐Golgi intermediate compartment. J Cell Biol. 1995;131(6 Pt 1):1403‐1419. 8522600 10.1083/jcb.131.6.1403 PMC2120650

[56] Zuber C , Fan JY , Guhl B , Roth J . Misfolded proinsulin accumulates in expanded pre‐Golgi intermediates and endoplasmic reticulum subdomains in pancreatic beta cells of Akita mice. FASEB J. 2004;18(7):917‐919. 15033933 10.1096/fj.03-1210fje

[57] Koumandou VL , Natesan SK , Sergeenko T , Field MC . The trypanosome transcriptome is remodelled during differentiation but displays limited responsiveness within life stages. BMC Genomics. 2008;9:298. 18573209 10.1186/1471-2164-9-298 PMC2443814

[58] Tiengwe C , Brown AE , Bangs JD . Unfolded protein response pathways in bloodstream‐form trypanosoma brucei? Eukaryot Cell. 2015;14(11):1094‐1101. 26318397 10.1128/EC.00118-15 PMC4621318

[59] Goldshmidt H , Matas D , Kabi A , Carmi S , Hope R , Michaeli S . Persistent ER stress induces the spliced leader RNA silencing pathway (SLS), leading to programmed cell death in Trypanosoma brucei. PLoS Pathog. 2010;6(1):e1000731. 10.1371/journal.ppat.1000731 PMC2809764 20107599

[60] Overath P , Engstler M . Endocytosis, membrane recycling and sorting of GPI‐anchored proteins: Trypanosoma brucei as a model system. Mol Microbiol. 2004;53(3):735‐744. 15255888 10.1111/j.1365-2958.2004.04224.x

[61] Huitema K , van den Dikkenberg J , Brouwers JF , Holthuis JC . Identification of a family of animal sphingomyelin synthases. EMBO J. 2004;23(1):33‐44. 14685263 10.1038/sj.emboj.7600034 PMC1271672

[62] Tafesse FG , Ternes P , Holthuis JC . The multigenic sphingomyelin synthase family. J Biol Chem. 2006;281(40):29421‐29425. 16905542 10.1074/jbc.R600021200

[63] Young SA , Smith TK . The essential neutral sphingomyelinase is involved in the trafficking of the variant surface glycoprotein in the bloodstream form of Trypanosoma brucei. Mol Microbiol. 2010;76(6):1461‐1482. 20398210 10.1111/j.1365-2958.2010.07151.x PMC2904498

[64] Schuck S , Simons K . Controversy fuels trafficking of GPI‐anchored proteins. J Cell Biol. 2006;172(7):963‐965. 16567497 10.1083/jcb.200603015 PMC2063753

[65] Sutterwala SS , Hsu FF , Sevova ES , et al. Developmentally regulated sphingolipid synthesis in African trypanosomes. Mol Microbiol. 2008;70(2):281‐296. 18699867 10.1111/j.1365-2958.2008.06393.x PMC2629665

[66] Sutterwala SS , Creswell CH , Sanyal S , Menon AK , Bangs JD . De novo sphingolipid synthesis is essential for viability, but not for transport of glycosylphosphatidylinositol‐anchored proteins, in African trypanosomes. Eukaryot Cell. 2007;6(3):454‐464. 17220466 10.1128/EC.00283-06 PMC1828920

[67] Stanne TM , Kushwaha M , Wand M , Taylor JE , Rudenko G . TbISWI regulates multiple polymerase I (pol I)‐transcribed loci and is present at pol II transcription boundaries in Trypanosoma brucei. Eukaryot Cell. 2011;10(7):964‐976. 21571922 10.1128/EC.05048-11 PMC3147422

[68] Martin KL , Smith TK . The glycosylphosphatidylinositol (GPI) biosynthetic pathway of bloodstream‐form Trypanosoma brucei is dependent on the de novo synthesis of inositol. Mol Microbiol. 2006;61(1):89‐105. 16824097 10.1111/j.1365-2958.2006.05216.x PMC3793301

[69] Smith TK , Crossman A , Brimacombe JS , Ferguson MA . Chemical validation of GPI biosynthesis as a drug target against African sleeping sickness. EMBO J. 2004;23(23):4701‐4708. 15526036 10.1038/sj.emboj.7600456 PMC533043

[70] Sutterlin C , Doering TL , Schimmoller F , Schroder S , Riezman H . Specific requirements for the ER to Golgi transport of GPI‐anchored proteins in yeast. J Cell Sci. 1997;110(Pt 21):2703‐2714. 9427388 10.1242/jcs.110.21.2703

