# Supplementary material for: Blocking variant surface glycoprotein synthesis alters endoplasmic reticulum exit sites/Golgi homeostasis in Trypanosoma brucei
Source: Traffic. 2018 Apr 6;19(6):391–405. doi: 10.1111/tra.12561 (PMC6001540; doi:10.1111/tra.12561)
Supplement: Supplementary file 2 — Appendix S1 Supporting Information Figure S1 Morpholino anti‐sense oligonucleotides as a strategy to block VSG synthesis. A, Anti‐sense Morpholino oligonucleotides were designed to bind the area, including and downstream of the start codon (indicated with a box) of VSG221 or α‐tubulin mRNA. Oligonucleotides either targeted the WT sequence, or had mismatched nucleotides incorporated (indicated in red), which would disrupt oligonucleotide binding. B, Transfection of mismatched α‐tubulin or VSG221 Morpholinos does not result in phenotypic changes compared with untransfected cells, or Trypanosoma brucei VB1.1 where VSG221 RNAi has not been induced. Microscopy analysis is shown of cells 12 hours after Morpholino transfection or before induction of VSG RNAi visualised using differential interference contrast (DIC) or the DNA stain DAPI. The scale bar indicates 5 μM Figure S2 TbSec24.1::Ty1 co‐localises with TbSec23.2::HA in the ERES. A, Immunofluorescence microscopy images showing overlap of TbSec23.2::HA (green) with that of TbSec24::Ty1 (red). Cells were imaged using Differential Interference Contrast (DIC) with DAPI staining of DNA is shown in blue. Scale bar represents 5 μM. B, The signal intensity of TbSec23.2::HA and TbSec24.1::Ty1 along a linear line drawn through the centre of both signals (left panel) was measured (right panel). The displacement of both signals was defined by the sum in the distance of the red and the green signal at the start and end of the peaks. The mean displacement between the 2 signals was 0.03 ± 0.3 μM (n = 25 cells that were 1K1N, error in SD). A representative trace is shown Figure S3 The total number of cisternae per Golgi stack does not change significantly in cells where VSG synthesis has been blocked for 24 hours (h). A, Schematic showing a transmission electron microscopy (TEM) image of BSF Trypanosoma brucei where the relevant subcellular structures are indicated, with the endoplasmic reticulum (ER) indicated in yellow, the Golgi c [file TRA-19-391-s002.pdf]

Supplemental material for:

Blocking Variant Surface Glycoprotein synthesis alters ERES/ Golgi homeostasis in *Trypanosoma brucei*

Cher-Pheng Ooi, Terry K. Smith, Eva Gluenz, Nadina Vasileva Wand, Sue Vaughan and Gloria Rudenko

Comprising:

Supplementary figure S1: Morpholino anti-sense oligonucleotides as a strategy to block VSG synthesis.
This contains control experiments for the Morpholino experiments.

Supplementary figure S2: Colocalisation of TbSec24.1 and TbSec23.2 in the ERES.
This immunofluorescence microscopy data confirms that both Sec proteins indeed colocalise.

Supplemental figure S3: The total number of cisternae per Golgi stack does not change significantly in cells where VSG synthesis has been blocked for 24 hours (h)
This figure contains additional analyses of the EM data.

Supplementary figure S4: Quantitation of *T. brucei* metabolic labelling experiments whereby the incorporation of radioactive labelled precursors into whole cells (uptake), total protein or lipids was followed after blocking VSG synthesis for various periods.
This figure contains additional control analyses for the metabolic labelling experiments.

Supplementary figure S5: Parent-ion scanning of the collision induced fragment for choline-phosphate (m/z 184) by positive ion ESI-MS-MS showing phosphatidylcholine (PC) and sphingomyelin (SM) phospholipids of lipid extracts from *T. brucei*.
This figure contains additional control data for the lipid synthesis analyses.

Supplementary Table 1: Lipid composition of *T. brucei* VG1.1 cells in the presence or absence of the induction of VSG RNAi for 24 hours.
This figures contains a detailed list of the lipid composition of trypanosomes in the presence or absence of a VSG synthesis block.

A

VSG221

start

WT 5'-GCCGGGCCTCCTGATTGGAAGGCAT-3'

Mismatch 5'-GCCCGGCATCCTCATTGCAAGGGAT-3'

tubulin

start

WT 5'-TGTGGATGCAGATAGCCTCACGCAT-3'

Mismatch 5'-TGTGCATGCACATACCCTGACGGAT-3'

B

DIC **DAPI**

No transfection

Mismatch αTubulin morpholinos (20 μM)

Mismatch α-VSG221 morpholinos (80 μM)

VB 0 h VSG221 RNAi

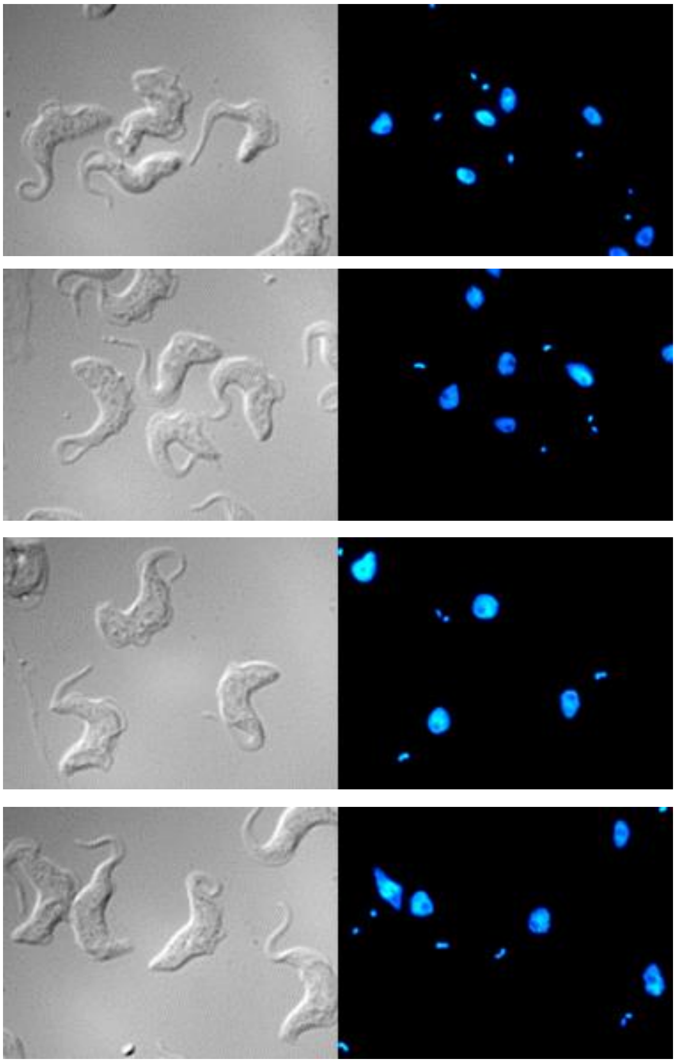

Sup Fig S1

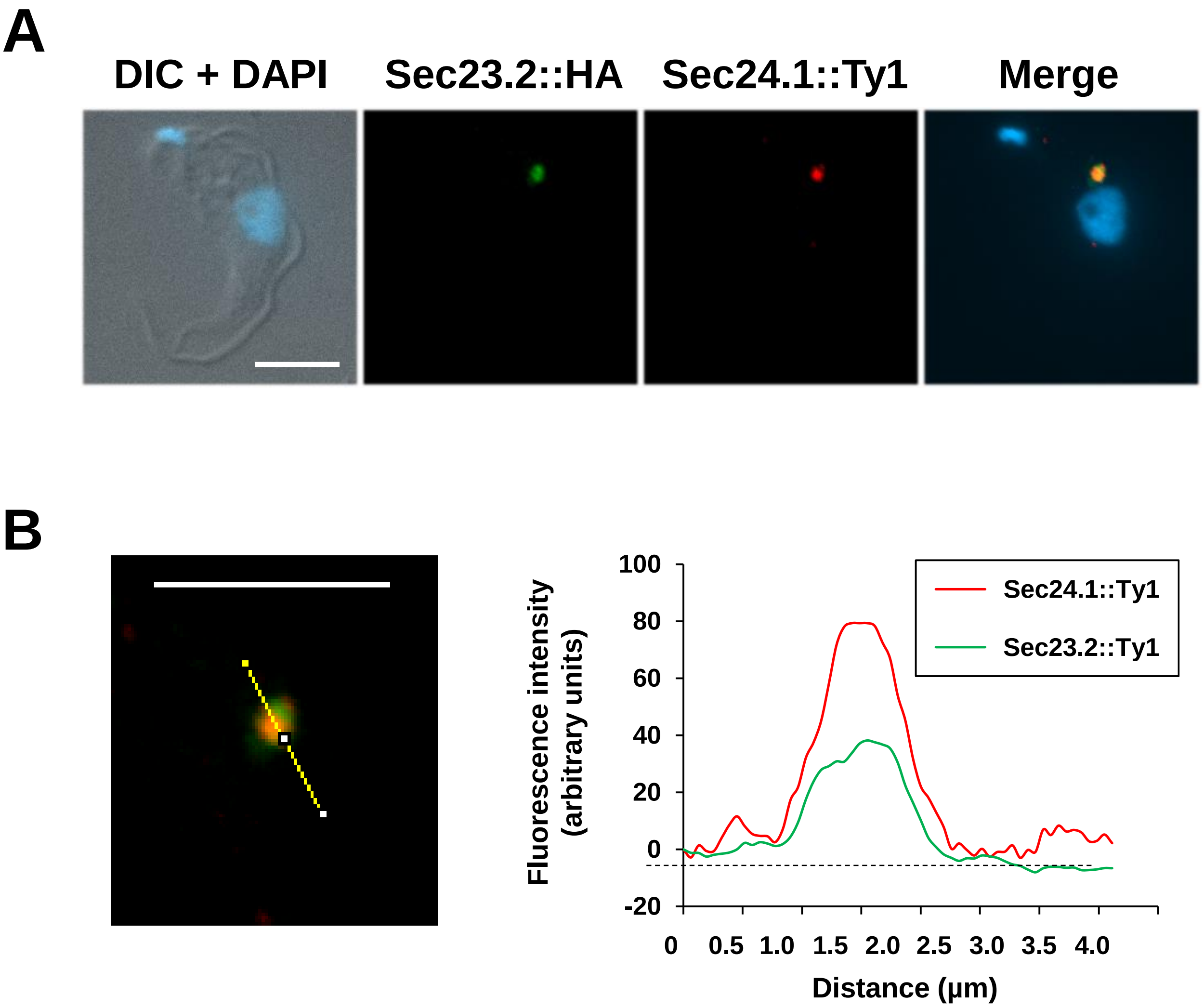
A
DIC + DAPI
Sec23.2::HA
Sec24.1::Ty1
Merge
B
Fluorescence intensity (arbitrary units)
100
80
60
40
20
0
-20
Sec24.1::Ty1
Sec23.2::Ty1
0
0.5
1.0
1.5
2.0
2.5
3.0
3.5
4.0
Distance (µm)

Sup Fig S2

A

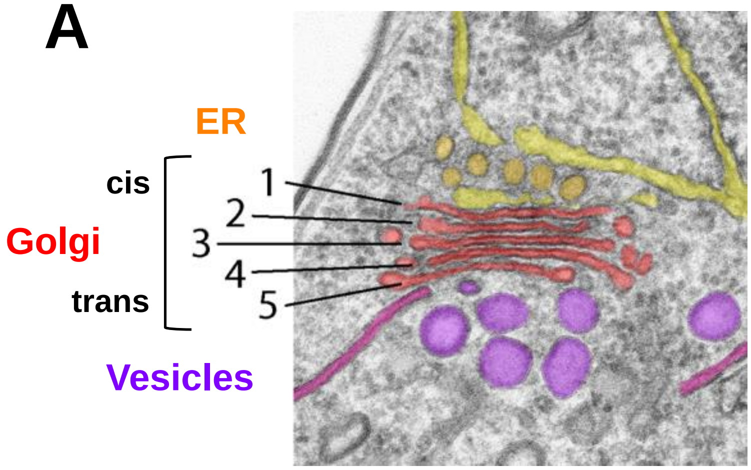
ER
cis
Golgi
trans
1
2
3
4
5
Vesicles

B

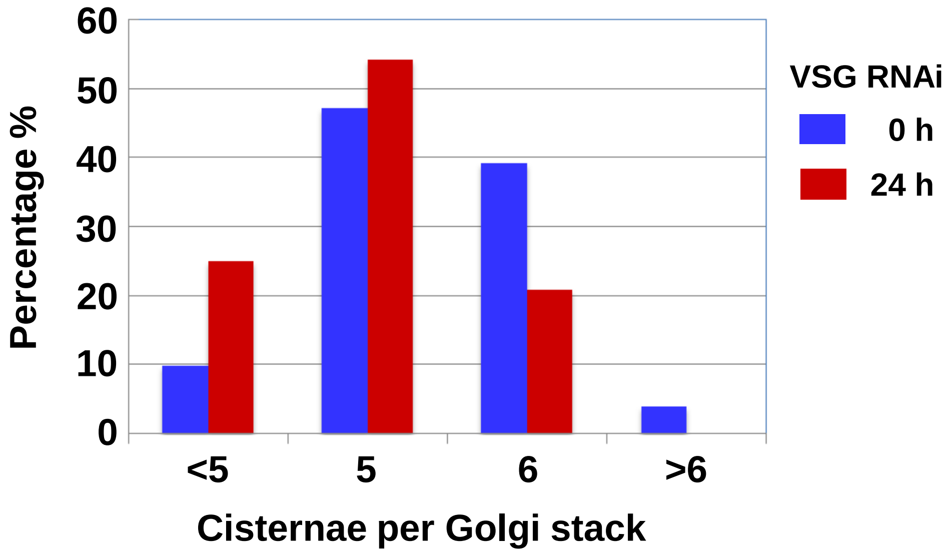
60
50
40
30
20
10
0
Percentage %
<5
5
6
>6
Cisternae per Golgi stack
VSG RNAi
0 h
24 h

Sup Fig S3

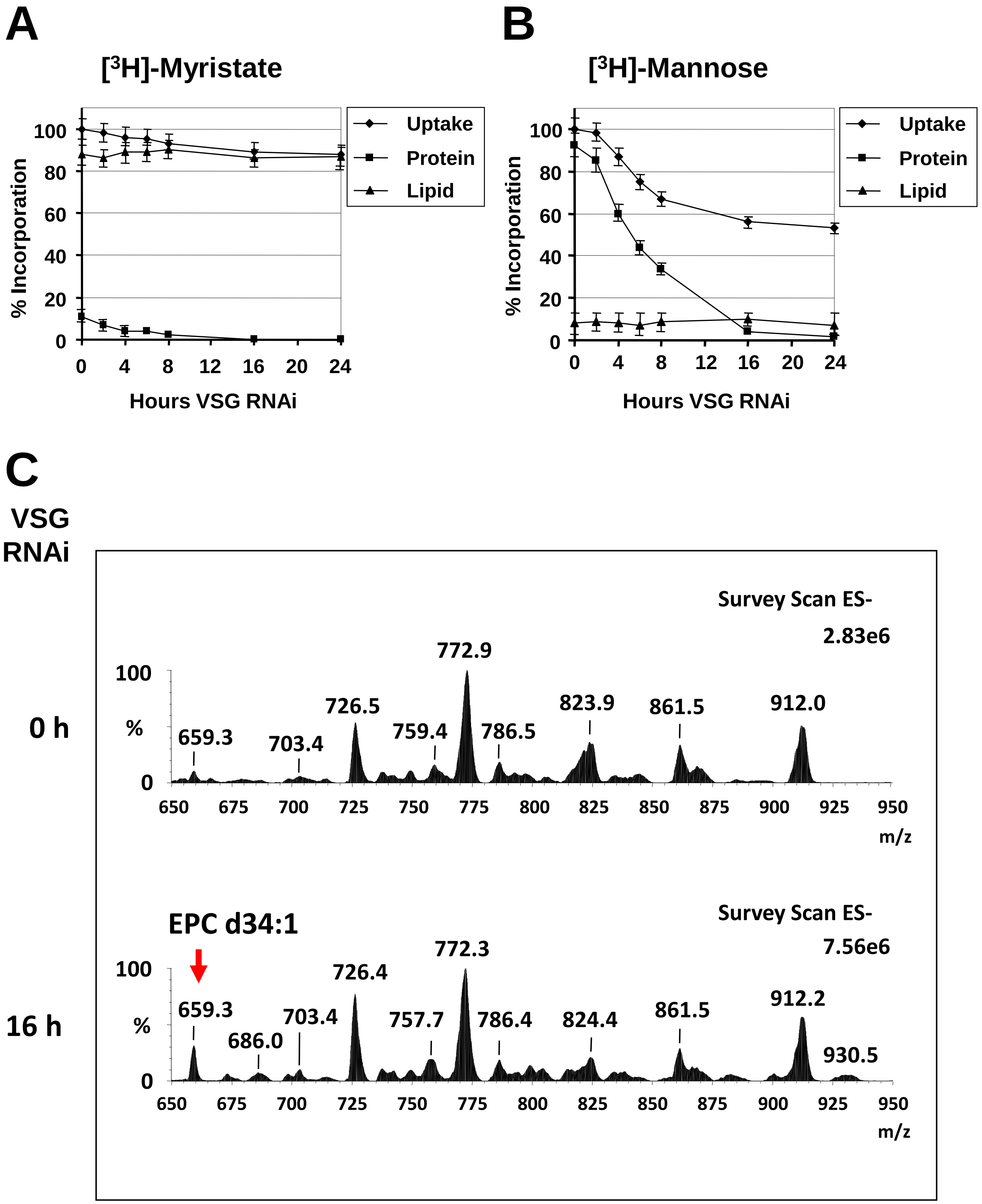
A
[3H]-Myristate
% Incorporation
Hours VSG RNAi
Uptake
Protein
Lipid
B
[3H]-Mannose
% Incorporation
Hours VSG RNAi
Uptake
Protein
Lipid
C
VSG RNAi
0 h
Survey Scan ES-
2.83e6
772.9
726.5
823.9
861.5
912.0
659.3
703.4
759.4
786.5
m/z
16 h
EPC d34:1
Survey Scan ES-
7.56e6
772.3
726.4
659.3
686.0
703.4
757.7
786.4
824.4
861.5
912.2
930.5
m/z

Sup Fig S4

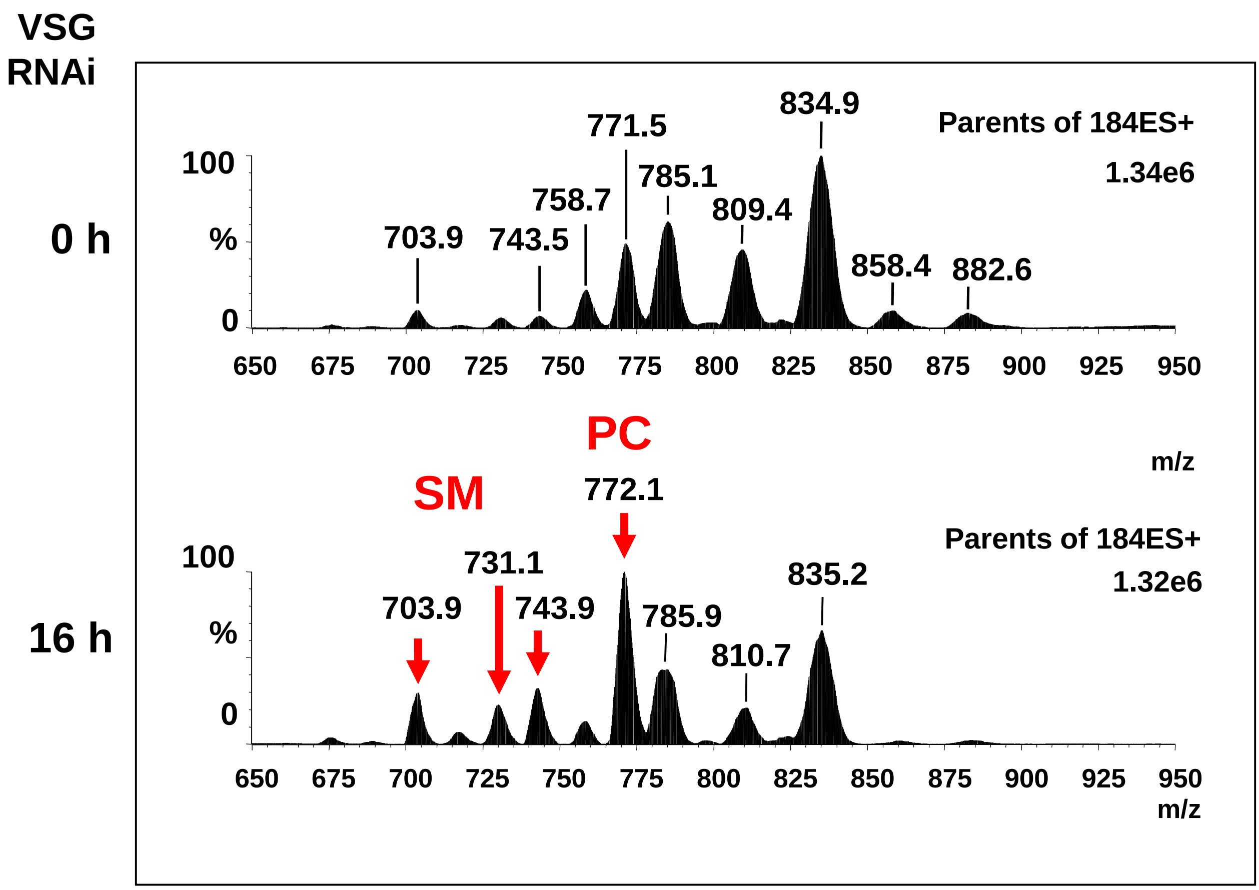
VSG
RNAi
0 h
Parents of 184ES+
1.34e6
100
%
0
834.9
771.5
785.1
758.7
809.4
703.9
743.5
858.4
882.6
650
675
700
725
750
775
800
825
850
875
900
925
950
m/z
16 h
PC
SM
772.1
Parents of 184ES+
1.32e6
731.1
703.9
743.9
785.9
810.7
835.2
m/z

Sup Fig S5

Supplementary Table 1

Lipid composition of *T. brucei* VG1.1 cells in the presence or absence of the induction of VSG RNAi for 24 hours.

m/z range	Principal Component	*m/z*	RNAi (-)	RNAi (+)
698-706	Sphingomyelin d32:1	703	3.5 %	8.5 %
714-720	Plasmalogen 32:0	717	0.5	1.5
728-734	Sphingomyelin d34:1	730	1.5	5.5
738-748	Plasmalogen 34:2	744	1.5	7.5
754-762	Sphingomyelin d36:1	758	5.5	1.5
766-776	Plasmalogen (38:2)	772	14.5	26.5
778-790	Diacyl 36:2 (18:0, 18:2)	786	19.0	20.5
806-818	Diacyl 38:4 (18:0, 20:4)	810	13.5	5.0
828-846	Diacyl 40:5 (18:0, 22:5)	836	32.5	18.0
852-864	Diacyl 42:8 (20:4, 22:4)	858	2.5	0.5
878-902	Diacyl 44:9 (22:5, 22:4)	884	2.5	0.5

Precursor scanning for the 184 m/z ion detects phosphocholine containing lipids such as phosphatidylcholine (PC) and sphingomyelin (SM).
Peak identities refer to the spectrum in Sup Fig 3C.
[M+H]+ ions, defined here are the principal component within a series, with the greatest intensity within brackets.
