# Supplementary material for: Blocking variant surface glycoprotein synthesis alters endoplasmic reticulum exit sites/Golgi homeostasis in Trypanosoma brucei
Source: Traffic. 2018 Apr 6;19(6):391–405. doi: 10.1111/tra.12561 (PMC6001540; doi:10.1111/tra.12561)
Supplement: Supplementary file 1 — Editorial Process [file TRA-19-391-s001.pdf]

Title: **Blocking Variant Surface Glycoprotein synthesis alters ERES/ Golgi homeostasis in *Trypanosoma brucei***

Authors: **Cher-Pheng Ooi, Terry K. Smith, Eva Gluenz, Nadina Vasileva Wand, Sue Vaughan and Gloria Rudenko**

Article Type: Original Research

Monitoring Editor Dominique Soldati-Favre
Date Submitted 20 September 2017
Date for Decision 1 12 November 2017
Date Resubmitted 2 February 2018
Date for Decision 2 27 February 2018
Accepted 8 March 2018

Decision and Reviews

Dear Dr. Rudenko, dear Gloria,

Thank you for submitting your manuscript "Blocking Variant Surface Glycoprotein synthesis alters ERES/ Golgi homeostasis in Trypanosoma brucei" for consideration for publication in Traffic. I asked two colleagues who are experts in the field to review the paper and their verbatim comments are appended below. The referees share the view that the work presented in this paper will be of interest to the readers of Traffic, but that some additional experiments and quantification is required before the paper can be accepted. In particular, I believe that the electron microscopy analysis recommended will significantly strengthen your conclusions. In addition, referee 2 notes that you will need to be more thorough in your citation of the literature, in particular the literature related to ER stress.

Although I cannot accept your manuscript for publication at this point, I believe that you will be able to address the referees' concerns and I look forward to receiving your revised manuscript. To expedite handling when you resubmit please be sure to include a response outlining how you have addressed each of the referees' concerns.

Sincerely,

Dominique Soldati-Favre, Ph.D.
Associate Editor
____
Referee's Comments to the Authors

Referee: 1

Comments to the Author
This paper reports the interesting finding that blocking the translation of the main cell surface protein (VSG) in parasitic trypanosomes results in precytokinesis arrest and affects Golgi homeostasis. The Rudenko lab has previously shown that ablation of the VSG mRNA results in a similar phenotype. Until now, however, it was unclear if the effect was due to a shortage of VSG mRNA or protein. This question is now solved. Additionally, the trypanosome reacts to VSG depletion by reducing the number of ERES/Golgi, supporting the notion that the flux of proteins feeds back on the architecture of the secretory system.

The manuscript is of high quality and originality. The morpholino approach is convincing and the quantification of ERES/Golgi numbers is conclusive, although the 2-digit errors suggest a depth of analysis that is not justified. This should be corrected.

The description of the distortion of ER and Golgi is clean, however, the ER-IFAs could be improved. How do the authors actually define the term 'dilated'?

That vesicular trafficking is unaffected (despite distortion of ER) appears odd. The authors may consider to elaborate on this a bit more. What exactly is a 'modest' reduction? Provide numbers for Hsp70 and TbCatL.

Besides the above minor points, the manuscript is well suited for publication in Traffic.

Referee: 2

Comments to the Author
The study from the Rudenko group is expanding our knowledge on the very interesting phenomenon described by their group that blocking VSG expression results in arrest of parasite growth and at a very precise cell cycle stage before cell division.

The study focuses on one very important essential question in understanding this phenomenon -what triggers this signaling pathway is it the reduction in mRNA level or the reduction in the protein.

The data presented nicely demonstrate using morpholino oligonucleotides which specifically block translation, that it is the block in protein synthesis rather than the reduction in the level of the mRNA which triggers the cellular consequences of the VSG depletion. This is a very important aspect of this work.

The study did not go beyond this very important observation to tell us how the block in protein synthesis leads to the affect on cell cycle. This is the key question but not easy to answer at this point an this study did not go in this direction.

The study focused on how the block in the synthesis of VSG affects the vesicular transport (ER to Golgi and the Golgi). It also looked at changes in lipdomics under VSG depletion and found no effect on on lipid metabolism except change in sphinomyelin which was increased. This is an important observation and the data is very convincing.

The study showed reduction in the number of ER exit sites by a the localization of Sec24.1 (Fig. 2 and 3). The also showed changes in the ER morphology (Fig. 5). I am very much convinced based on their data that decrease in the bulk of proteins that need to get to intra-vesicular system, decrease the amount of exit sites.
I will be more convinced if the study would show EM immunogold data and also showing COPII ...

If the VSG is not made and there is no load on the ER why would the ER be dilated?

It will be more convincing to show BiP in the ER by immuno-gold.

The work also suggest that that silencing of VSG does not affect bulk intra-cellular ...indeed no affect was seen for the biogenesis of lysosomal cathespin.
I wanted to point out to the authors that GPI anchored protein may have different machinery of exit and even intracellular trafficking compared to membrane proteins or secreted proteins ...as these protein utilize in trypanosomes and yeast the SRP-independent pathway for translocation across the ER.

I think that the paper has very interesting observation to advance our understanding of VSG block pathway but I would like to see the trafficking issues demonstrated in higher resolution that presented and as detailed above.

Minor comments

The Figures 2 and 3 describes very quantitavely the phenomenon of the number of exist site and number of Golgi and hence it is not necessary to describe in details the number and S.D in the text the many details makes it hard to follow.

ER stress has been studied extensively in the trypanosomes by several groups and I am surprised to see the authors decided only to cite studies from other systems.

Author Rebuttal

Dear Prof. Soldati, Dear Dominique,

We would like to resubmit the manuscript “Blocking Variant Surface Glycoprotein synthesis alters ERES/ Golgi homeostasis in Trypanosoma brucei” by Ooi et al for reconsideration by Traffic. We found that the referees made some very helpful comments on our manuscript, and have responded to their concerns with additional experiments and modifications to the text in the manuscript.

With regards to analysis of BiP: we have now performed additional immunofluorescence microscopy experiments analysing BiP expression in the presence of a VSG synthesis block throughout the cell cycle. We include these new images in a new Figure 5.

With regards to analysis of the ERES, extensive immunogold experiments have localised TbSec23.2 to the ERES (Sevova and Bangs, 2009). In contrast, we have used the less well characterised TbSec24.1 as an ERES marker. We have now generated a new BF T.

brucei cell line where both Sec proteins have been epitope tagged in the same cell. TbSec24.1 is tagged with the Ty1 epitope and TbSec23.2 with the HA epitope. We show that TbSec23.2 and TbSec24.1 indeed colocalise. This agrees with data from Sevova and Bangs, 2009, although we have performed more extensive measurements of the co-localisation. These data are now included in a new Supplemental Figure S2.

As extensive immunogold data is already available for TbSec23.2, we do not see the added value of performing additional immunogold experiments for a colocalised protein. This is particularly as we never argue in this manuscript that localisation of these proteins changes after the induction of a VSG synthesis block. Similarly, we do not see that there is added value to looking at an ultrastructural level at BiP, as here too we do not argue that its location changes in any way. We very much hope that you will be flexible on this issue.

We have now rectified omissions in the previous version of the manuscript including reference to different characterised ER stress responses in T. brucei. In addition, we have rectified other mistakes or issues that were unclear. For example, we have now clarified that we find evidence for a 'distorted' rather than a 'dilated' ER.

We feel that our manuscript is now much improved. We would like to thank the referees for their thoughtful feedback, and hope that you now agree with us that our manuscript is now suitable for publication in Traffic. We look forward to hearing from you regarding our submission.

Sincerely,

Gloria

Decision and Reviews

Dear Dr. Rudenko, dear Gloria,

Thank you for submitting your revised manuscript "Blocking Variant Surface Glycoprotein synthesis alters ERES/ Golgi homeostasis in Trypanosoma brucei" to Traffic. I asked referee 2 to read the revised paper. The referee did not include comments for the author, but indicated in confidential comments that you have addressed the concerns raised previously. I agree with the referee and I am pleased to accept this paper for publication.

Sincerely,

Dominique

Dominique Soldati-Favre, Ph.D.
Associate Editor
